# Structural Analysis of the SARS‐CoV‐2 Spike N‐Terminal Domain Across Wild‐Type and Recent Variants: A Comparative Study

**DOI:** 10.1002/prot.26855

**Published:** 2025-06-09

**Authors:** Miriana Quaranta, Allegra Via, Stefano Pascarella

**Affiliations:** ^1^ Department of Biochemical Sciences “A. Rossi Fanelli” Sapienza University of Rome Rome Italy

**Keywords:** 4A8, electrostatic surface, flexibility, interaction energy, molecular dynamics, NTD, SARS‐CoV‐2

## Abstract

Since its first appearance in China, the molecular evolution of SARS‐CoV‐2 has progressed through altering the properties of the Spike protein, changing the virus ability to transmit and to evade host immune surveillance. Despite receiving less attention than the Receptor Binding Domain (RBD), the Spike N‐Terminal Domain (NTD) is crucial to SARS‐CoV‐2 biology and pathogenesis. This study provides a comparative structural analysis of the NTD from the wild‐type strain and different variants (BA.2, XBB.1, XBB.1.5, BA.2.86, JN.1, HV.1, KP.2, KP.3, and KP.3.1.1), aiming to clarify the structural impact of mutations in each variant. To assess the impact of mutations on the interaction of NTD with antibodies, we selected as a test case the neutralizing antibody 4A8, which has proven highly effective against the WT. The results obtained from molecular dynamics simulations, surface electrostatic potential analysis, and binding energy predictions show a clear trend in the evolution of the virus. The net charge of the NTD decreases as the variants progress, reaching a minimum charge of −1.84 observed for KP.3.1.1. This is in clear contrast to the RBD net charge, which follows an opposite trend toward higher positive values. Binding energy predictions show that the antibody's efficacy decreases as the virus evolves. While the WT exhibited an interaction energy of −96.28 kcal/mol with 4A8, more recent variants like KP.3 show no interaction stronger than −64.00 kcal/mol. These results reveal a clear trend of modifications aimed at favoring immune escape in the virus' evolutionary trajectory.

## 1 Introduction

SARS‐CoV‐2, an RNA virus with a single‐stranded, positive‐sense genome, exhibits a high error rate during RNA replication, leading to mutations that can impact virus susceptibility to neutralizing antibodies and transmissibility [1]. The continuous evolution of the SARS‐CoV‐2 virus since its first appearance in China [2, 3] has presented an ever‐shifting landscape in the battle against the COVID‐19 pandemic. For example, the omicron variant (BA.1.1.529) [4] has represented a turning point in the evolution trajectory of SARS‐CoV‐2 for its relatively high number of mutations and deletions observed in the Spike protein. Since then, a myriad of variants with different degrees of concern have arisen, as for example, the BA.2.86 variant, also known as Pirola, which appeared in late July 2023 [5]. After Pirola, several other variants emerged via mutations or recombination events, with different capacities of diffusion including XBB.1.5 [6, 7], JN.1 [8], HV.1, [9] and the most recent KP.2 [10], KP.3 [11] and KP.3.1.1 [12]. More will inevitably appear in the future.

The emergence of SARS‐CoV‐2 variants has had, and is still having, a significant impact on global public health. In this context, to implement effective containment strategies, an in‐depth understanding of the structural and functional differences of the variants is necessary. These needs are reflected in the vast deluge of studies published in scientific literature aimed at delineating the evolutionary trajectory of SARS‐CoV‐2 and understanding the functional impact of the Spike protein molecular remodeling. Much emphasis has been given to the Receptor Binding Domain (RBD) as it is involved in the interaction with the main cellular virus receptor, the angiotensin‐converting enzyme 2 (ACE2) receptor, and displays several epitopes targeted by both monoclonal antibodies and the host immune system [13, 14]. Much less, attention has been paid to the Spike N‐terminal domain (NTD), although it plays important roles in SARS‐CoV‐2 biology and pathogenesis: for example, it may influence virus tropisms, host immune response; it interacts with the RBD contributing to the recognition of secondary receptors and co‐receptors, and can be a drug target [15, 16].

This study provides a comparative structural analysis of the SARS‐CoV‐2 NTD from the wild‐type strain and the nine variants BA.2, XBB.1, XBB.1.5, BA.2.86, JN.1, KP.2, KP.3, and KP.3.1.1. Structural variations are examined, and potential implications for viral behavior and vaccine efficacy are discussed.

**FIGURE 1:**
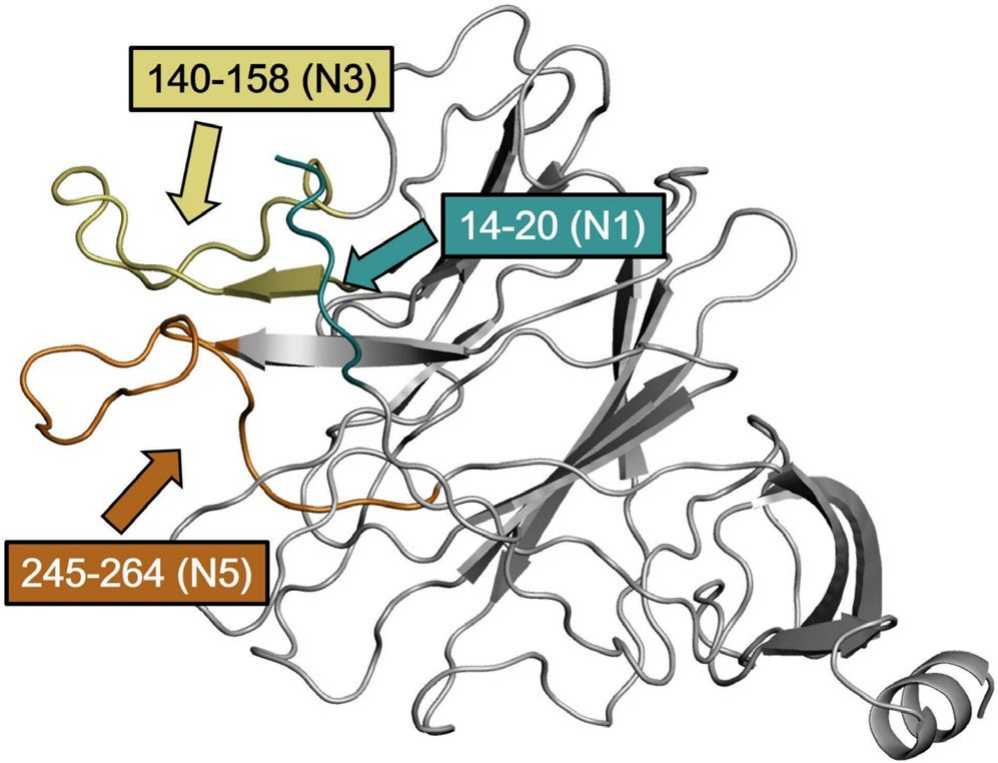
Cartoon representation of the NTD. The loops encompassed by residues 14–20 (N1), 140–158 (N3) and 245–264 (N5) are represented with cyan, yellow and orange colors, respectively.

By comparing the NTD structures across these different variants, we offer insights into the molecular mechanisms underlying the observed phenotypic differences and propose a foundation for future research and vaccine development strategies. We have also studied how the NTD mutations can impact the domain interaction with antibodies, using as a test case the neutralizing monoclonal antibody 4A8 [17]. This antibody was isolated from COVID19 patients and proved to possess high neutralization potency against SARS‐CoV‐2. Crystallographic studies revealed that the monoclonal antibody binds exclusively to the SARS‐CoV‐2 NTD. This observation prompted researchers to consider NTD as a target for therapeutic monoclonal antibodies (mAbs) [17]. Through structural and biophysical analyses of several mAbs bound to the Spike protein, an antigenic supersite on the NTD was identified [18], which is the target of 4A8. The supersite comprises loops encompassed by sequence positions 14–20, 140–158, and 245–264 (Figure 1) referred to as N1, N3, and N5, respectively. Two additional loops, designated as N2 and N4, are located at sequence positions 69–71 and 177–186.

The structural and functional impacts of NTD mutations have been investigated using a set of structural bioinformatics techniques, mainly based on molecular dynamic simulations and structural analyses. Besides the immediate application to human health, examining the molecular evolutionary trajectory of SARS‐CoV‐2 is of utmost interest as it contributes to unveiling principles of broad application in protein evolution and monitoring emergence of new, potentially dangerous variants. An in‐depth comprehension of the characteristics of these variants holds the potential to provide crucial insights into the potential trajectory of SARS‐CoV‐2, thereby informing the development of adaptive strategies to mitigate its impact on global public health. Through this analysis, we seek to contribute valuable data to the ongoing efforts to monitor and counteract the development of SARS‐CoV‐2 variants.

## 2 Materials and Methods

### 2.1 Structural Modeling

Sequences of SARS‐CoV2 variants BA.2, XBB.1, XBB.1.5, BA.2.86, JN.1, HV.1, KP.2, KP.3, and KP.3.1.1 were retrieved from the GISAID database [19]. The sequence of the wild‐type Spike was retrieved from the Uniprot [20] database (accession code P0DTC2). The complete list of Spike mutations and their prevalences was obtained from the outbreak.info website [21]. Potential effects on the NTD N‐glycosylation induced by variant mutations have been predicted using the program NetNGlyc 1.0 [22]. Homology models of variant Spike NTDs and RBDs were built with the program Modeler 10.4 [23]. The templates used for NTD and RBD modeling were the coordinate sets denoted by the PDB codes 7B62 and 6M0J, respectively. The same approach has been used to build the NTD‐4A8 antibody complexes, using as a template the PDB structure 7C2L. Spike structure visualization and analysis were performed using the graphic programs PyMOL [24] and ChimeraX [25].

### 2.2 Molecular Dynamics Simulation

Molecular dynamics (MD) simulations were carried out following standard procedures [26]. Calculations were performed using the GROMACS v. 2023.5 program [26, 27] along with the force field AMBER99SB‐ILDN [28]. The molecules were placed in a dodecahedral box, solvated with TIP3P water molecules, and positioned at a 1.5 nm distance to the box edge. The solvated system was neutralized to a final concentration of 0.15 M NaCl. The system was minimized with the steepest descent minimizer until convergence, namely, until no change in energy between successive steps was detected or until the maximum force was less than 10.0 kJ ^−1^mol^−1^ nm^−1^. After minimization, the system was subjected to 2 ns of NVT and 2 ns of NPT equilibration at 300 K with a modified Berendsen thermostat (time constant 1 ps). Production simulations were carried out using the NPT ensemble. The LINCS algorithm [29] was applied to constrain the bond lengths. Electrostatic forces were calculated with the Particle Mesh Ewald method [30] using a grid spacing of 0.16 nm. A cutoff of 1.0 nm was set for short‐range electrostatic and van der Waals interactions. The production simulation was run for 1 μs in the case of isolated NTD domains and for 500 ns for the NTD‐4A8 complexes. To assess system stability, one independent replica of 1 μs and 500 ns simulation of the NTD and NTD‐4A8 complex of each variant was calculated, respectively. A shorter trajectory was calculated for the RBDs of the same variants to assess possible fluctuation of the net charge in order to compare it with NTDs. The time step was set to 2 fs. Trajectories were visualized with the software VMD 1.9.3 [31] and analyzed with the GROMACS tools combined with the XMGRACE graphic program [32].

### 2.3 Structural Analysis

A 100 snapshots of the NTD and RBD molecular dynamic trajectories were taken, respectively, and the net charge at pH = 7.0 was calculated with the software PROPKA3 [33]. The final net charge of each domain was the average calculated on the extracted snapshots. In fact, PROPKA3 determines the pKa of an ionizable group by applying an environmental perturbation to the unperturbed pKa value of the group. These perturbations might change slightly during the simulation, inducing a temporary change in the pKa of the ionizable groups and the net charge of the protein [33].

NTD electrostatic potential surface has been mapped with the aid of the SURFMAP 2.2.0 software [34]. SURFMAP implements a method of “molecular cartography” by which a protein three‐dimensional surface can be projected onto a two‐dimensional plane to analyze and compare the distribution of specific physico‐chemical properties. Electrostatic maps have been calculated for each snapshot of the trajectory for every domain taken as described above. The final map displaying the electrostatic surface of each domain was defined as the average map calculated over 100 snapshot maps with the aid of R script run within the R‐Studio environment [35].

Interaction energy of the complexes NTD‐4A8 was estimated using the method implemented in the program gmx_MMPBSA [36]. The analysis was conducted on the last 250 ns of the 500 ns dynamic simulations using a sampling interval of 1 ns. Residues located within 4 Å of the NTD‐4A8 interface providing a significant energetic contribution were identified using the per‐residue effective free energy decomposition (prEFED) protocol [37] available in the gmx_MMPBSA software. The van der Waals energies (ΔEvdw), electrostatic energies (ΔEele) and internal terms (ΔEint) were calculated using the ff19SB force field [38]. The solvation free energies were computed by calculating polar components using the Poisson‐Boltzmann procedure, setting the dielectric model on 2 (ipb = 2) [39]; the nonpolar component was estimated using the surface area (SA) method [40]. The Interaction Entropy method was used to determine the entropic contribution [41].

The conformations adopted by the NTD‐4A8 complexes during the simulations were clustered using the *gmx cluster* function in GROMACS, along with the gromos clustering algorithm [42].

The R package Bio3d 3.0 [43] was used to generate correlation matrices of the motions of Cα atoms over the last 250 ns of each simulation. The dynamics cross‐correlation network was calculated for the NTD domain of all variants isolated from the simulations of the NTD‐4A8 complexes.

## 3 Results

### 3.1 Molecular Modeling

**FIGURE 2:**
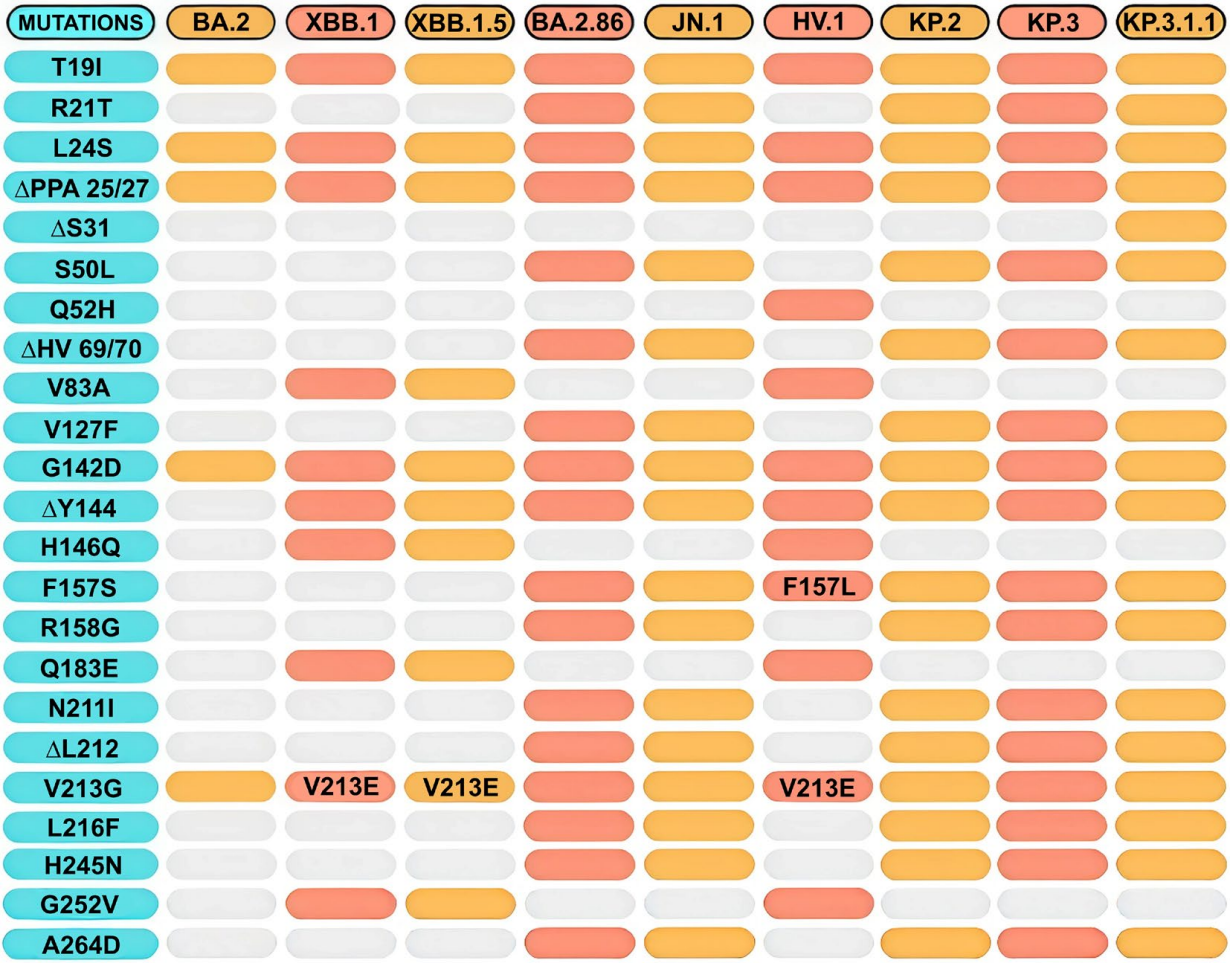
List of NTD mutations. All the variants analyzed are listed in the header. Blue cells indicate all the mutations. Red and orange cells indicate the presence of the mutation in the corresponding variant while gray cells indicate absence. Mutations differing from the reference reported in the blue column are explicitly indicated within the cells.

In this study, the NTDs of the original virus strain and the variants BA.2, XBB.1.5, BA.2.86, HV.1, and KP.3.1.1 have been compared. Figure 2 reports the mutation characterizing each variant with respect to the reference original strain. It should be noted that the three recent variants JN.1, KP.2, and KP.3 have the same identical mutations on NTD as BA.2.86. Therefore, observations made on NTD for the BA.2.86 variant can be extended to JN.1, KP.2, and KP.3. Similarly, NTD displays identical mutations in the XBB.1 and XBB.1.5 variants. Therefore, similar considerations apply to the domain of these two variants.

In addition, the interaction of BA.2, XBB.1.5, BA.2.86, and HV.1 NTDs with the monoclonal antibody 4A8 has been simulated to shed light on how the virus evolution affects the Spike affinity for antibodies and assess the potential impact of specific NTD mutations.

Although the experimental structures of several Spike variants are now deposited in the PDB, the structure of the loop encompassed by sequence positions 245–264 (corresponding to N5) is missing and, therefore, the domains necessitate reconstruction. A further example is represented by the missing coordinates of loops encompassed by sequence positions 72–77 (N2), 179–186 (N4) and 250–255 (N5) in the crystallographic structures of the complex NTD‐4A8 for the BA.2 variant (PDB: 8DM3 and 8DM4). In cases like these, the domain necessitates reconstruction. To ensure standardized starting conditions for subsequent structural analyses and, most importantly, for comparison purposes, we therefore decided to reconstruct the entire NTD for all variants using the same model.

**FIGURE 3:**
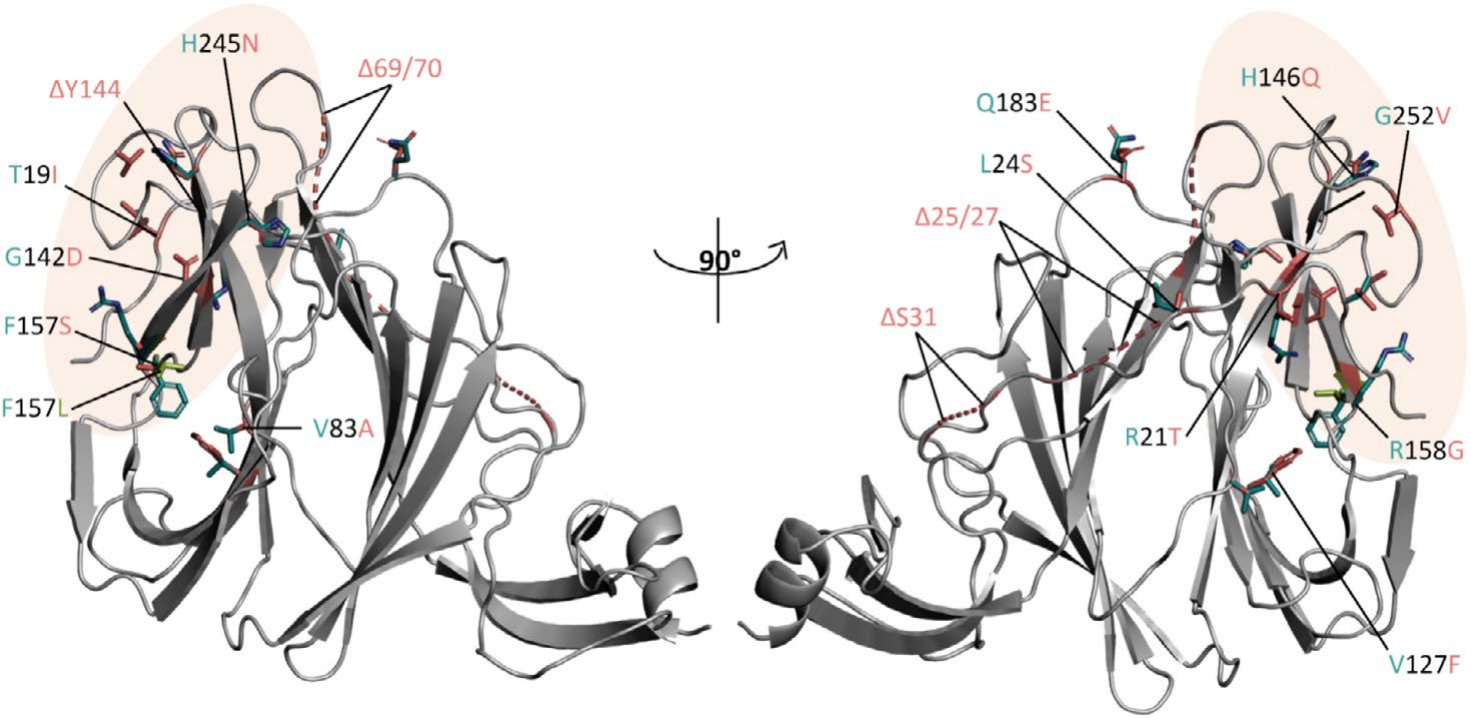
NTD structure represented with cartoon models. The orange ovals indicate the NTD interface with the antibody 4A8. Mutant side chains are represented as stick models and labeled with one‐letter code where cyan color denotes the original residue. The deletions are indicated by pink dashed lines and labeled by the greek letter ‘Δ’.

Figure 3 reports the distribution of variant mutations within the NTD structure and in relation to the interface with 4A8. The single mutation specific to KP.3.1.1 (ΔS31) is located far from the 4A8 binding site. Indeed, S31 is located at 4.5 nm from residue 252, which marks the “central” position of the NTD interface to the antibody. For this reason, the complex KP3.1.1 NTD‐4A8 has not been modeled, assuming that the influence of the mutation on the 4A8 binding site is negligible.

In general, progressive accumulation of mutations can be observed on the NTD domain from the early to the latest variants (Figure 2). Five mutations are present in BA.2 NTD while 18 are observed in KP.3.1.1. Interestingly, four mutations in BA.2 (T19I, L24S, Δ25/27, G142D) are shared by all the variants considered in this work. There are other differences: V213 is replaced by Glu in XBB.1, XBB.1.5, and HV.1, while it is substituted by Gly in the other variants. F157 is replaced by Leu only in HV.1.

Potential effects of mutations on NTD N‐glycosylation sites were explored. The prediction of N‐glycosylation sites (Table S1) recognizes the presence of eight sites on the wild‐type (WT) NTD, which were previously determined experimentally: N17, N61, N74, N122, N149, N165, N234, N282 [44]. N‐linked glycosylation is characterized by the Asn‐X‐Ser/Thr motif, which is recognized by oligosaccharyltransferase during protein synthesis in the endoplasmic reticulum, where the N‐glycan is attached to the asparagine residue. Our analysis highlighted the loss of the N17 glycosylation site in all variants [45, 46] caused by the emergence of the T19I mutation (Figure 2). In fact, the I19 substitution disrupts the N‐linked glycosylation consensus sequence. Interestingly, in BA.2.86, JN.1, KP.2, KP.3, and KP.3.1.1 a new glycosylation site appeared at position 245 as the result of the H245N mutation. This can be experimentally observed in the BA.2.86 Spike structure with PDB code 8Y4A. In KP.3.1.1, an additional glycosylation site has been predicted at position N30 (Figure 2 and Table S1). Indeed, the deletion of S31 leads to the formation of the consensus sequence “Asn‐Phe‐Thr‐Arg”, which can be recognized by oligosaccharyltransferase [9].

### 3.2 Molecular Dynamics

Molecular dynamics trajectories for the NTD domains of the Spike variants under study, each spanning 1 μs, were computed. The complexes NTD‐4A8 were simulated for 500 ns, a simulation time sufficient to assess the interaction energy with the program gmx_MMPBSA [36]. To reduce the complexity of the simulated molecular systems, glycan substituents were excluded from the models.

Simulation equilibrium was assessed by monitoring the variation of the principal physico‐chemical properties such as temperature, pressure, and energy with simulation time (data not shown). Structural stability of the domains and complexes during molecular dynamics simulation was monitored by the variation of the backbone Radius of Gyration and Root Mean Square Deviation (RMSD) with respect to the initial structure versus simulation time (Figure S1).

**FIGURE 4:**
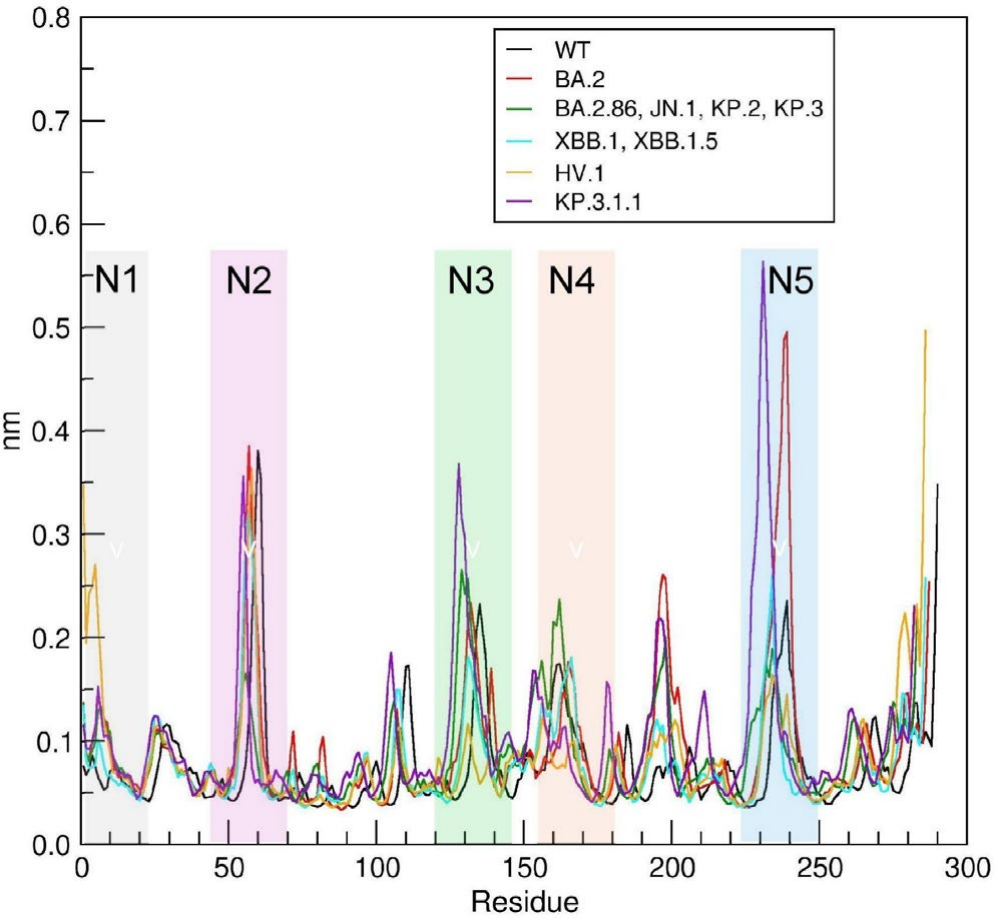
Backbone Root Mean Square Fluctuation (RMSF) of the variant NTDs. Positions of the N1, N2, N3, N4, and N5 loops are indicated by the colored transparent boxes with labels.

Differences in local chain flexibility were studied by comparison of the Root Mean Square Fluctuation (RMSF) profiles. The RMSF was calculated over the last 800 ns of the 1 μs simulated trajectory to assure that the systems were at equilibrium and stable. The RMSF plot (Figure 4) indicates that the loops are intrinsically flexible and that the variant mutations do not alter significantly the flexibility of loops N1, N2, and N4. In contrast, the flexibility of N3 and N5 is affected by the mutations [47].

### 3.3 Structural Analysis

**FIGURE 5:**
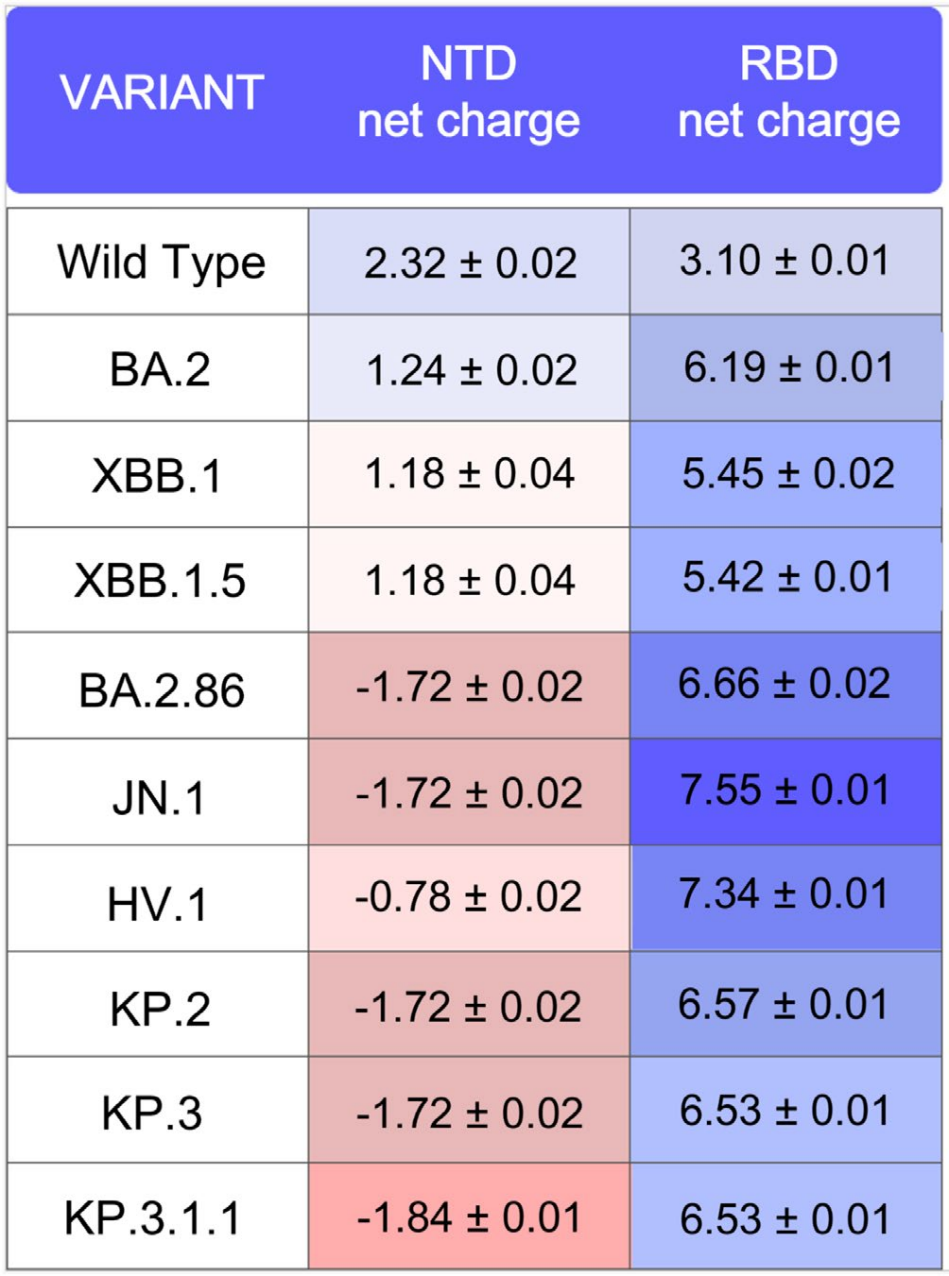
Comparison of net charges of NTD and RBD in the wild‐type and the other variants. Cell background colors indicate the intensity and the sign of the corresponding charge.

Macromolecule surface electrostatic potential is pivotal in influencing interactions with receptors and other cellular components [48, 49, 50]. In the NTD, alterations in the net charge may indicate changes in intra‐ or inter‐molecular interactions across different variants. The NTD net charge was calculated with the program PROPKA3 [33] as a quantitative description of the dominant charge of surface electrostatic potential (Figure 5) by sampling the molecular dynamics trajectory as described in the Material and Methods section to detect charge fluctuations over time. For comparison purposes only, the net charge of the corresponding RBDs was calculated using the same method and a 300 ns molecular dynamics simulation.

Comparison of the variant NTD net charge with the original SARS‐CoV‐2 strain suggests that, in the most recent variants, the charge has been changing toward progressively more negative values. The net charge of the wild‐type NTD is +2.32 ± 0.02, and this value gradually decreased from 1.24 ± 0.02 in BA.2 up to −1.84 ± 0.01 in KP.3.1.1. This pattern is clearly opposite to what is observed in the evolution of the RBD, where the net charge has been increasing across variants toward more positive values. The trend toward increasingly negative values is driven by mutations such as R21T, R158G, ΔH69, H245N, A264D in variants BA.2.86, JN.1, KP.2, KP.3, and KP.3.1.1, leading to loss of positive or gain of negative charge. The mutation Q183E in variants XBB.1, XBB.1.5, and HV.1 led to the increment of negative charge.

Accordingly, the distribution of the charge on the surface has also changed. Comparison of the two‐dimensional projections of the surface potential is reported in Figure S2, which displays the progressive alteration of the surface charge distribution.

### 3.4 NTD—Antibody Complex

Potential impact of the NTD mutations on the interaction with antibodies has been assessed using as a test case the complex between 4A8 and the NTDs of different variants. Cluster analysis was applied to group the trajectories of each complex into four clusters, with cluster 1 containing the most populated conformation (Figure S3). The number of clusters was set on the basis of the clusterization of the wild‐type NTD. In this case, the application of a cut‐off corresponding to the median of the RMSD distribution curve resulted in the identification of four main conformational groups. The interaction energies calculated using gmx_MMBPSA based on the molecular dynamics simulations are summarized in Table 1. The role of the individual heavy (4A8H) and light (4A8L) chains of 4A8 in the interaction with NTD has also been studied in detail.

**TABLE 1:** Interaction energy (kcal/mol) between the antibody 4A8, its light (4A8L) and heavy (4A8H) chains, and the NTD variants.

	WT	BA.2	XBB.1	BA.2.86	HV.1
			XBB.1.5	JN.1	
				KP.2	
				KP.3	
4A8	−96.28 ± 0.93	−84.69 ± 1.03	−92.50 ± 1.32	−64.00 ± 1.07	−90.64 ± 1.10
4A8H	−91.56 ± 0.84	−83.59 ± 1.05	−81.39 ± 1.14	−63.37 ± 1.06	−84.54 ± 1.05
4A8L	−6.33 ± 0.27	−0.40 ± 0.08	−11.40 ± 0.51	−0.28 ± 0.03	−7.82 ± 0.25

#### 3.4.1 Wild‐Type Complex

**FIGURE 6:**
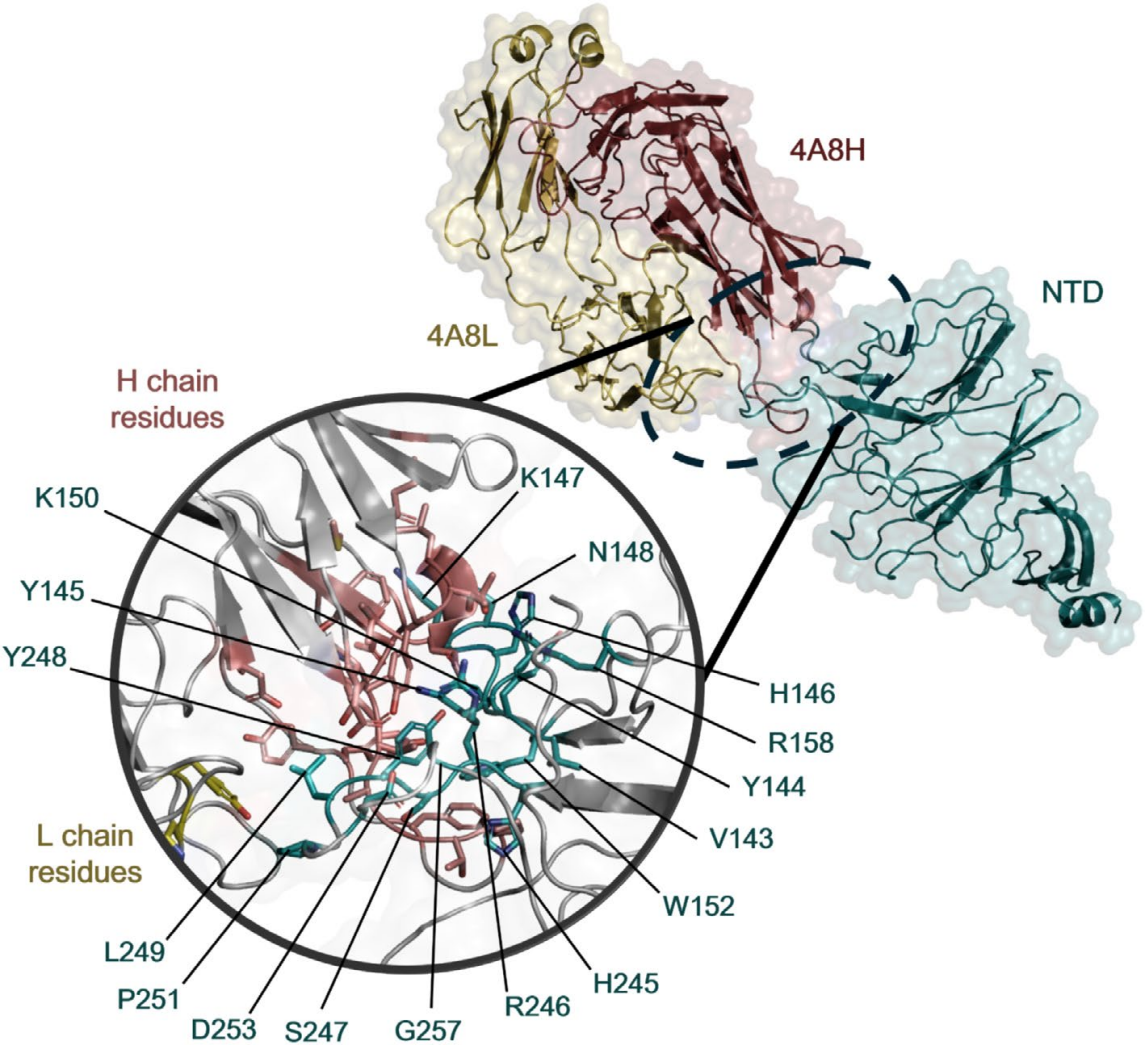
Structural representation of the interface between the WT NTD and 4A8. The NTD is depicted in cyan and the residues involved in the interaction are shown as sticks. The heavy chain of 4A8 is highlighted in red salmon‐red, its interacting residues displayed as sticks. The light chain of 4A8 is shown in yellow, and the residues involved in the interaction are represented as sticks.

**FIGURE 7:**
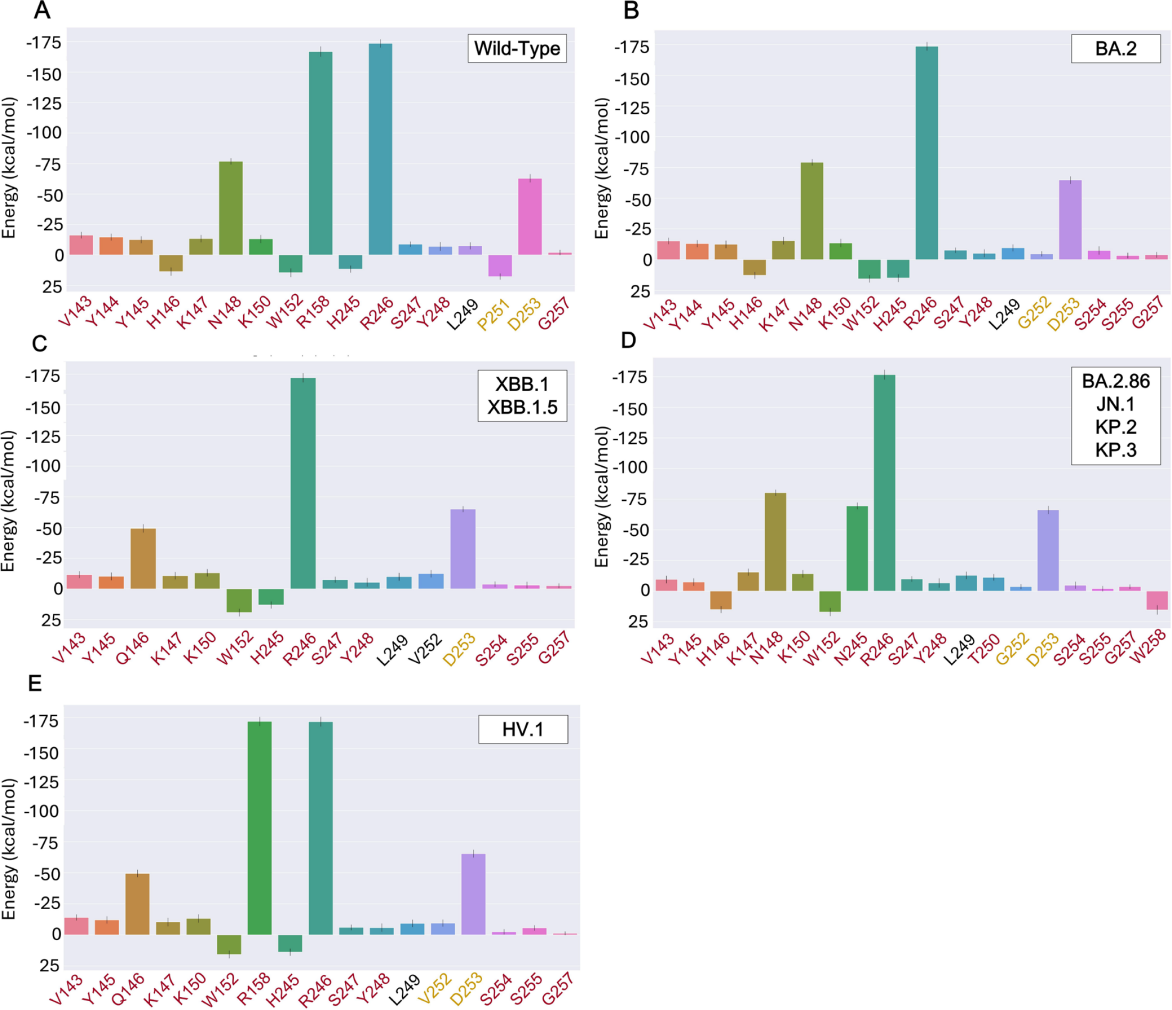
Total energy decomposition contributions (TDC) of the NTDs residues forming the interface with 4A8 in the different variants: (A) wild type; (B) BA.2; (C) XBB.1 and XBB.1.5; (D) BA.2.86, JN.1, KP.2 and KP.3; (E) HV.1. Salmon‐red, yellow and black labels on the x‐axis denote the residues interacting with the heavy, light and both chains, respectively. The Y‐axis represents the energy in kcal/mol.

Figure 6 displays the interface between the WT NTD and 4A8 as defined by McCallum et al. 2021 [18]. The NTD interface for the 4A8 antibody is composed of 17 residues (Figure 7A and Table S2) belonging to loops N1, N3, and N5. However, it has been shown that only N3 and N5 contribute significantly to the interface [17]. Sixteen of these residues contact the Complementarity Determining Regions (CRD) CDR1, CDR2, and CDR3 of the heavy chain of 4A8, while only two residues interact with 4A8L. L249 is the only residue that interacts with both chains.

**FIGURE 8:**
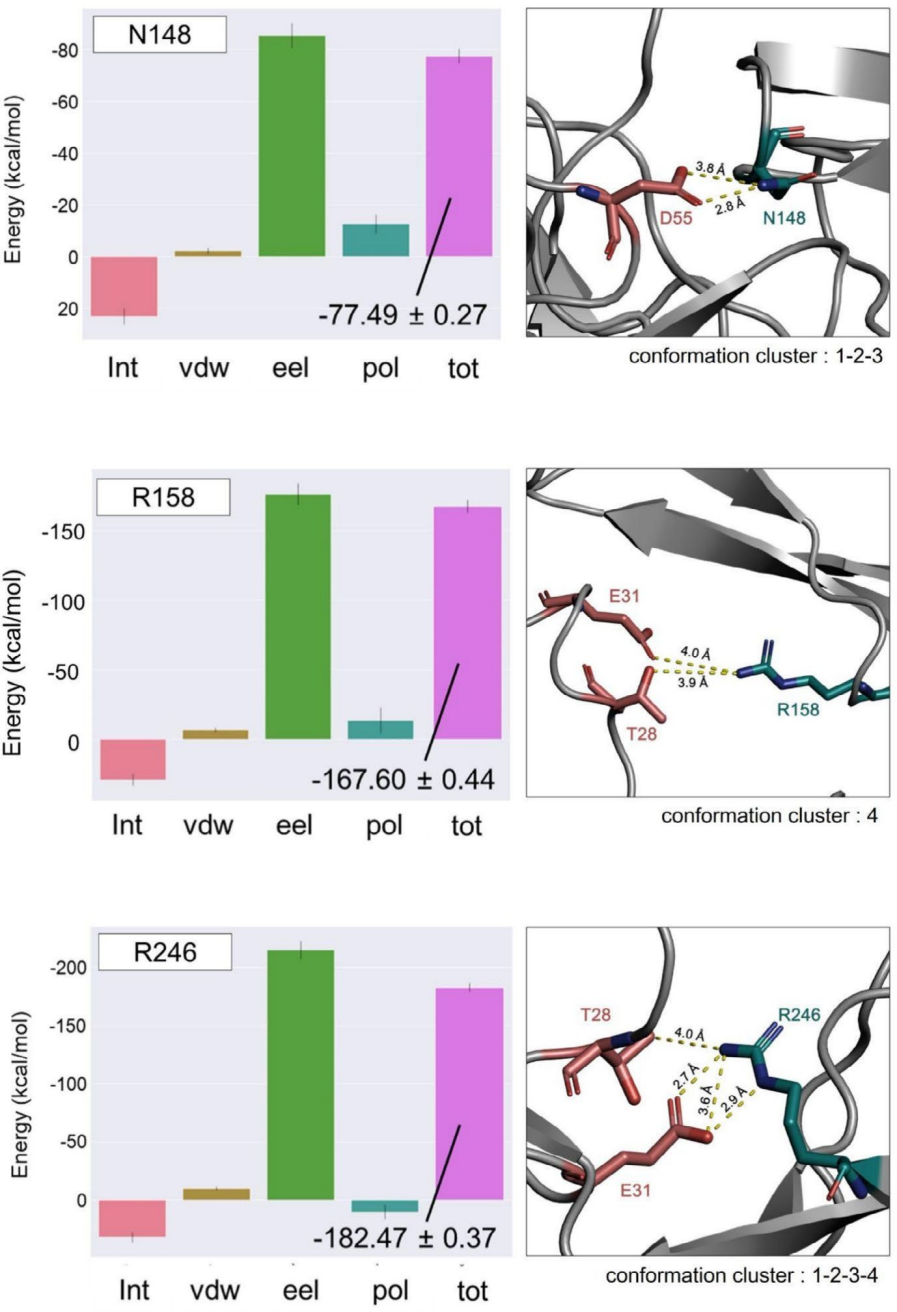
Bar plots of the decomposition of energy components (kcal/mol) of the WT NTD residues N148, R158 and R246 which contribute most to the stability of the interaction. Int = Internal energy contributions; vdw = van der Waals energy contributions; eel = electrostatic energy contributions; pol = polar solvation free energy contributions; tot = total free energy contributions (sum of all). At the side of the bar plots, the structural representation of residue interactions is displayed. Cyan and salmon‐red labels indicate NTD and 4A8H residues, respectively. Distances between residues are shown as dotted lines labeled with the corresponding distance value.

**FIGURE 9:**
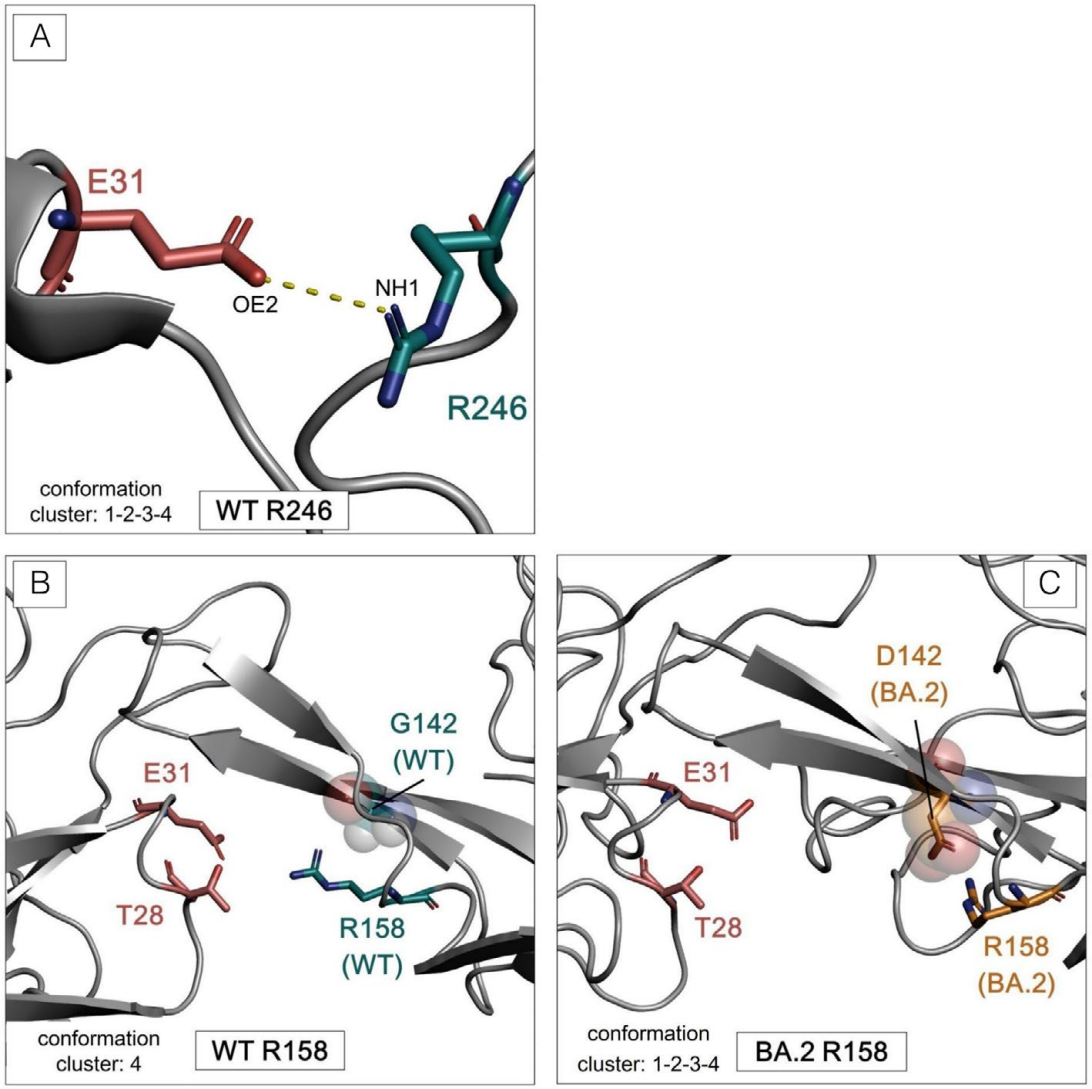
Specific interactions at the interface with 4A8. (A) Salt bridge between NH1 of R246 residue of the WT (cyan stick) and OE2 of E31 residue of 4A8H (salmon‐red stick). The yellow dotted line indicates the distance between the two atoms equal to 2.8 Å. (B) Comparison of the conformations of the loop containing R158 in the WT (cyan stick) and in (C) BA.2 (orange stick) 4A8H residues are shown in salmon‐red sticks. The WT residue G142 and BA.2 D142 are shown as cyan or orange sticks respectively inside transparent spheres to indicate steric occupancy.

Three of the NTD residues that interact with 4A8H, N148, R158, and R246, provide the highest contribution to the interface stability (Figure 7A and Figure 8). The residue that plays the largest role in stabilizing the light chain interaction is D253, but the overall contribution of this chain is less relevant compared to the heavy chain. R246 contributes the most to the interaction energy, with a value of −182.47 ± 0.37 kcal/mol in its contact with 4A8H. In fact, this residue interacts with the antibody in every cluster detected (Figure S3). The most significant component of the interaction energy of the three residues is electrostatic (eel) (Figure 8, Table S3, Figure S4). Residue R246 can establish a salt bridge with E31 of the heavy chain of 4A8 (Figure 9A). In contrast, NTD residues H146, W152, H245, and P251 have marginal destabilizing effects (Figure 7A, Table S3).

#### 3.4.2 Variant Complexes

The interaction energy (Table 1) predicted for the BA.2 NTD‐4A8 complex is higher than that of the wild‐type complex and lower than that of more recent variants. Compared to WT, the BA.2 interaction with the light chain plays a less prominent role. Although no BA.2 mutation occurs within the supersite interacting with 4A8, the energy contribution of the residues to the interface stability changes (Figure 2, Figure 7B). In BA.2, 18 residues interact with 4A8 (Figure 7B, Table S2). However, the simulation shows that the interactions mediated by R158 with the heavy chain and P251 with the light chain are lost, while two interactions with the heavy chain mediated by S254 and S255 and one with the light chain made by G252 are gained (Figure 7B). Overall, the residues that contribute most significantly to the stabilization of the interaction between BA.2 and 4A8 are N148 and R246, as in the WT (Figure 7B, Table S3).

XBB.1 and XBB.1.5 NTDs are identical. The interaction energy is higher than the WT and lower than all the variants (Table 1). The interface with 4A8 is composed of 16 residues (Figure 7C and Table S2). Two interface residues are mutated (H146Q, G252V) and one deleted (ΔY14). Mutation H146Q provides an indirect stabilizing interaction (Figure 7C, Table S3, Figure S5) by removing the destabilizing effect of H146, and this situation is observable in all four clusters (Figure S3). The stabilizing interactions mediated by residues N148 and R158 are also lost. Residue V252, which does not form interactions in the NTD WT and only interacts with 4A8L in BA.2, in this variant interacts with both antibody heavy and light chains.

The NTD sequences of BA.2.86, JN.1, KP.2, and KP.3 are identical. The interaction energy with 4A8 is among the highest observed (Table 1). The interface with 4A8 is composed of 19 residues (Figure 7D and Table S2). The mutations occurring at the interface are R158G, which abolishes the interaction made by R158, and H245N (Figure 7D and Table S3) which introduces a stabilizing interaction with 4A8H (Figure 7D) and creates a new glycosylation site (Table S1). This site is reported in the structure present in the PDB with the code 8Y4A. The deletion of Y144 obliterates all the interactions made by this residue. Interactions made by P251 are lost, while a new interaction is mediated by the residue T250 (Table S2). As in the BA.2 NTD, G252 interacts only with the light chain.

HV.1 NTD displays an interaction energy similar to XBB.1 and XBB.1.5 (Table 1). The interface with the antibody is composed of 17 residues (Figure 7E, Table S2). Unlike all the other variants analyzed, HV.1 possesses the unique mutations Q52H and F157L. The mutations H146Q and G252V occur at the 4A8 interface. The effect of H146Q is similar to that observed in the variants XBB.1 and XBB.1.5. In HV.1, the V252 residue interacts only with 4A8L, whereas in XBB.1 and XBB.1.5 it interacts with both chains. Nevertheless, this residue provides an energy contribution to the HV.1—4A8 complex that is almost identical to that of the XBB.1 and XBB.1.5 variants (Figure 7C,E).

## 4 Discussion

While many studies have been devoted to the characterization of RBD variants, similar attention has not been paid to NTD. Indeed, RBD plays a crucial role in the viral entry into host cells. It specifically binds to the ACE2 receptor on human cells, facilitating the virus' attachment and subsequent fusion with the host membrane. This interaction is a crucial step in the infection process and plays a key role in the virus's pathogenicity. Additionally, the RBD is a major target for neutralizing antibodies, making it a focus for vaccine development and therapeutic interventions. However, the NTD of the Spike protein of SARS‐CoV‐2 also plays several key roles in the viral life cycle. Moreover, the role of the spike NTD has also been described in other coronaviruses [51]. Primarily, it is involved in mediating the initial stages of viral entry by facilitating interactions with host cell receptors [52] and possibly with co‐receptors like AXL or sialic acids [53] thus influencing tissue tropism. Additionally, it has been implicated in evading the host immune response, as it can act as a target for neutralizing antibodies [54].

In this work, a comparative analysis of the NTDs of a set of recent SARS‐CoV‐2 variants stemming from BA.2 has been conducted. In particular, the wild‐type NTD has been compared to BA.2 and a selection of descendant variants that have been under the attention of the World Health Organization: XBB.1, XBB.1.5, BA.2.86, JN.1, HV.1, KP.2, KP.3, and KP.3.1.1. Mutations have accumulated in the NTD as the variants have progressed (Figure 2). The occurrence of these modifications led to interesting changes in the structural properties of Spike's NTD. These changes affect Spike's functional properties, including interaction with antibodies and immune escaping ability. To investigate how mutations can influence the interaction with antibodies, the interaction with the 4A8 antibody was taken as a test case in this study.

The Spike protein of SARS‐CoV‐2 is known to be glycosylated by the host cell machinery, resulting in glycans that are defined as “self” [55]. Glycans are known for their ability to sterically block interaction sites, preventing antibody binding [56]. They also tend to mask the entire surface of epitopes, making them harder for antibodies to recognize; this effect is so pronounced that it is often called the “glycan shield” [55, 57]. This mechanism can be observed in action in the variants' NTDs analyzed in this study. In fact, the WT has eight glycosylation sites, while KP.3.1.1 is predicted to have nine glycosylation sites [44, 45] (Table S1). Furthermore, a rearrangement in the distribution of these sites driven by the emergence of mutations has been observed. In all the variants considered, the loss of the N17 site was observed [46] caused by the T19I mutation (Figure 2) that disrupts the consensus sequence for N‐linked glycosylation. In addition, the deletion of S31 in KP.3.1.1 is predicted to introduce another glycosylation site at position N30 (Figure 2 and Table S1), by creating the typical N‐linked glycosylation consensus sequence “Asn‐Phe‐Thr‐Arg” [9].

To simplify the molecular systems as much as possible, also taking into account the heterogeneity and the presence of different glycoforms in vivo [58, 59], glycans were omitted during the simulations. In a comparative context, the omission equalizes all the simulated systems and focuses on the impact of mutations on polypeptide chain properties. However, this omission can be a limitation of the present study.

The comparison of the NTD net charge (Figure 5), taken as a proxy of the surface electrostatic potential, shows that it has been steadily decreasing from the wild‐type to the most recent NTD variants in which negative values are observed. This trend is opposite to what is observed for the RBD variants. The surface electrostatic potential is considered one of the most important driving forces in the interaction between macromolecules and ligands [60]. Changes to this important structural property necessarily alter the interaction of the domain with other cellular components such as the negatively charged sialic acids [61, 62, 63] and can affect the virus behavior. Moreover, the modification of the electrostatic surface potential influences the interplay between the NTD and RBD within the Spike protein [64].

The modification of surface electrostatic potential certainly also influences the interaction with 4A8 as it is demonstrated by the increase of the binding energy from the original strain up to the recent variants (Table 1). The modification of the electrostatic potential is mediated by mutations of specific charged residues: for example, R21T, R158G, Q183E, and A264D remove positively charged residues (first two substitutions) and add negatively charged residues (last two).

**FIGURE 10:**
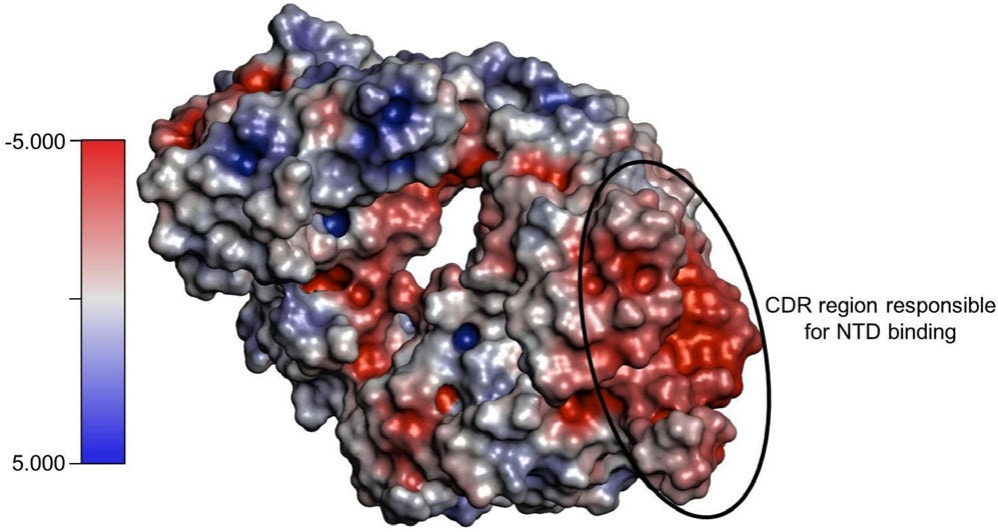
4A8 electrostatic surface visualization obtained with PyMOL APBS Electrostatics Plugin. The region encircled by an oval is the interface of 4A8 that makes contact with the NTDs. Electrostatic potential color scale is displayed where red and blue represent negative and positive charge, respectively. The charge is expressed as kT/e units.

It is noteworthy that the net charge of 4A8 is negative, with a PROPKA3 predicted value of −2.79 (Figure 10). Furthermore, the 4A8 CDR region responsible for NTD binding exhibits the most negative surface potential. This evidence supports our findings, as the decrease in net charge observed with the progression of the variants (Figure 5) is associated with a weakening of their interaction with the 4A8 antibody (Table 1). Therefore, the decrease in net charge of the NTD suggests that it is the result of the immune escape strategy adopted by the virus.

Accordingly, the evaluation of the interaction energies (Table 1) shows that the WT NTD has a particularly stable interaction with the 4A8. This result is in agreement with the results that demonstrated that 4A8 displays strong neutralizing capacities against the original SARS‐CoV‐2 virus. However, the complex stability seems to decrease as the variants progress (Figure 2, Table 1) while mutations remodel the NTD surface.

We analyzed in detail the impact of the mutations on the interactions with 4A8. The wild‐type NTD residues N148, R158, and R246 play a major role in stabilizing the interaction with 4A8 mainly by means of electrostatic interactions. In fact, N148, R158, and R246 interact with D55, T28, and E31 of 4A8H, respectively (Figure 8, Figure S4). Residue N148 interacts with the antibody in three out of four conformation clusters, while R246 interacts with 4A8 in all four clusters (Figure 8, Figure S3). R158 appears to establish interaction with its partner residues only in the conformations belonging to cluster 4 (Figure 8). Other residues have a marginally destabilizing effect that is completely compensated by the stronger stabilizing interactions. The interaction energies calculated for the structural complexes of the variants (Table 1) reflect a weakening of the interaction with this antibody. This observation is in line with the evidence of a loss of neutralizing potency of 4A8 in the SARS‐CoV‐2 Omicron variants [65].

BA.2, one of three Omicron lineages first detected in November 2021 [66], already reduces the neutralizing effect of 4A8, even with a small number of NTD mutations (Figure 2). Indeed, several studies demonstrated that BA.2 shows increased immune evasion compared to the wild type [46, 65, 67]. The five mutations characterizing the BA.2 NTD can impair 4A8 binding despite being located far from the interface. Indeed, comparative analysis of the minimum distance between the NTD residue R158 and the 4A8 E31 over the 500 ns of molecular dynamics simulation indicates that it has the average value of 0.4 and 1.5 nm in the WT and BA.2 NTD, respectively (Figure S6A). This suggests that the mutations perturb the conformation of the BA.2 N3 loop and hinder the formation of the salt bridge between R158 and E31. Accordingly, the main evidence of altered interaction with the antibody is the loss of the R158 contribution (Figure 7B) caused by an increased distance of this residue from its interactors (Figure S6). Additionally, the G142D mutation may have played a role by introducing a salt bridge between R158 and D142 observable in all the BA.2 cluster conformations (Figure S3). This modification helps keep the Arg away from the interface with the antibody (Figure 9B,C). Indeed, it is possible to observe how, during the simulation time, G142 is significantly farther from R158 compared to when it mutates to D142 (Figure S6B). As a matter of fact, it had already been hypothesized that the presence of G142D mutation could contribute to the high immune escape of BA.2 [68]. Comparison of the cross‐correlation matrices (Figure S7) indicates that the correlated motions of the wild‐type NTD are different from those of BA.2, especially in the region between 113 and 163, which includes residues R158 and D142.

All the variants analyzed in this study originate from the common ancestor BA.2 and then followed two different paths [69]. XBB.1, XBB.1.5 (which share the same NTD), and HV.1 followed the same evolutionary path; in fact, their mutations (Figure 2) and interaction energy with 4A8 (Table 1) are similar. The high similarity of HV.1, XBB.1, and XBB.1.5 is reflected in the evident similarity of the respective cross‐correlation matrices (Figure S7). In fact, the characteristic mutations of HV.1 do not appear to alter significantly the atomic motions compared to XBB.1 and XBB.1.5. The relatively favorable interaction energies with 4A8 could also result from the small number of mutations that these variants have accumulated in NTD. Despite the H146Q mutation introducing a stabilizing interaction with 4A8 (Figure 7C,E; Figure S5; Figure S8A,B), the deletion of Y144 causes a change in the conformation of the N3 loop in all the detected clusters (Figure S3). This alteration of several interactions is shown in Figure S4 and Figure S6C. The impact of the deletion at position 144 is also evident in the correlation matrices (Figure S7) of the wild‐type compared to XBB.1, XBB.1.5 (as well as HV.1), showing a more pronounced difference in the region between residues 113–163. Certainly, what makes the antibody less effective against these variants is the loss of the interactions mediated by N148 (in XBB.1, XBB.1.5, and HV.1) and R158 (in XBB.1 and XBB.1.5).

The more recent variants analyzed, BA.2.86, JN.1, KP.2, and KP.3.1.1 (all sharing the same NTD sequence) behave differently. The interaction energies resulting from the MD simulations (Table 1) reflect a weak interaction with the 4A8 antibody. This result is consistent with previous studies that have shown that only 1%–2% of the 4A8 antibody can neutralize the BA.2.86 and JN.1 variants, in contrast to the 92% that can neutralize the WT [70]. The mutations that primarily distinguish these variants from the previous ones are R158G and H245N (Figure 7D, Table S2). In particular, H245N introduces a new glycosylation site (Table S1). This is a crucial aspect because the interaction of N245 with the antibody may be compromised by the presence of glycans. This would further contribute to weakening the neutralizing power of 4A8 against these variants. The different behavior of these variants compared to the wild type can also be observed in their cross‐correlation networks (Figure S7). In fact, the regions spanning residues 113–163 and 213–263, encompassing positions 158 and 245 respectively, show marked differences.

## 5 Conclusions

Structural analyses conducted on the NTD of the Spike protein of the variants BA.2, XBB.1, XBB.1.5, BA.2.86, JN.1, HV.1, KP.2, KP.3, and KP.3.1.1 revealed the trend of the virus' evolutionary trajectory. The accumulation of mutations progressively increases as the variants progress, and this is reflected in structural changes that affect the NTD properties and interfere with the action of antibodies. In fact, the emergence of additional glycosylation sites helps “hide” the protein from the host's immune system, while simultaneously interfering with antibody binding. Moreover, the change in the net charge sign of the NTD also appears to suggest an evasion mechanism against the 4A8 antibody, which also has a negatively charged interface and exhibits high neutralizing potency against the positively charged WT's NTD. A clear indication of the reduced affinity of 4A8 for contemporary variants is provided by the increasingly higher interaction energies. One of the interactions that provides a significant energetic contribution to the NTD‐4A8 complex is R158, which is lost in almost all variants due to emerging mutations. It would therefore appear that the virus, particularly in recent variants, tends to accumulate mutations in the NTD that enhance its immune evasion.

This study highlights the impact of mutations in the NTD of the SARS‐CoV‐2 Spike protein on viral properties, including antigenicity, immune evasion, and receptors' interactions. Our observations underscore the dynamic adaptability of the virus and provide insights into variant‐specific phenotypes. These observations emphasize the importance of continuous monitoring of NTD mutations to inform therapeutic and vaccine strategies in combating emerging SARS‐CoV‐2 variants.

## Author Contributions

**Miriana Quaranta:** conceptualization, investigation, validation, visualization, formal analysis, data curation, methodology, writing – review and editing, writing – original draft, software. **Allegra Via:** validation, writing – original draft, writing – review and editing, conceptualization. **Stefano Pascarella:** supervision, data curation, methodology, writing – review and editing, writing – original draft, formal analysis, conceptualization, software.

## Conflicts of Interest

The authors declare no conflicts of interest.

## Peer Review

The peer review history for this article is available at https://www.webofscience.com/api/gateway/wos/peer‐review/10.1002/prot.26855.

## Supporting information


**Data S1**.

## Data Availability

The data that support the findings of this study are available from the corresponding author upon reasonable request.
