# Supplementary material for: Structural Analysis of the SARS‐CoV‐2 Spike N‐Terminal Domain Across Wild‐Type and Recent Variants: A Comparative Study
Source: Proteins. 2025 Jun 9;93(11):1891–904. doi: 10.1002/prot.26855 (PMC12517252; doi:10.1002/prot.26855)
Supplement: Supplementary file 1 — Data S1. [file PROT-93-1891-s001.docx]

**Supporting information**

**Table S1**

**NTD N-glycosylation sites**

| **Variant** |  | **Sequence position^a)^** | | | | | | | | |
| --- | --- | --- | --- | --- | --- | --- | --- | --- | --- | --- |
| **WT** | N17 | X | N61 | N74 | N122 | N149 | N165 | N234 | X | N282 |
| **BA.2** | X | X | N61 | N74 | N122 | N149 | N165 | N234 | X | N282 |
| **XBB.1** | X | X | N61 | N74 | N122 | N149 | N165 | N234 | X | N282 |
| **XBB.1.5** | X | X | N61 | N74 | N122 | N149 | N165 | N234 | X | N282 |
| **BA.2.86** | X | X | N61 | N74 | N122 | N149 | N165 | N234 | N245 | N282 |
| **JN.1** | X | X | N61 | N74 | N122 | N149 | N165 | N234 | N245 | N282 |
| **HV.1** | X | X | N61 | N74 | N122 | N149 | N165 | N234 | X | N282 |
| **KP.2** | X | X | N61 | N74 | N122 | N149 | N165 | N234 | N245 | N282 |
| **KP.3** | X | X | N61 | N74 | N122 | N149 | N165 | N234 | N245 | N282 |
| **KP.3.1.1** | X | N30 | N61 | N74 | N122 | N149 | N165 | N234 | N245 | N282 |
|  |  |  |  |  |  |  |  |  |  |  |

1. “X” means absence of glycosylation

**Table S2**

NTD-4A8 Interface composition

| **Residue position** |  | **WT** | **BA.2** | **XBB.1 XBB.1.5** | **BA.2.86 JN.1**  **KP.2**  **KP.3** | **HV.1** |
| --- | --- | --- | --- | --- | --- | --- |
| V143 |  | ✓ | ✓ | ✓ | ✓ | ✓ |
| Y144 |  | ✓ | ✓ | ✕ | ✕ | ✕ |
| Y145 |  | ✓ | ✓ | ✓ | ✓ | ✓ |
| H146 |  | ✓ | ✓ | ✕ | ✓ | ✕ |
| Q146 |  | ✕ | ✕ | ✓ | ✕ | ✓ |
| K147 |  | ✓ | ✓ | ✓ | ✓ | ✓ |
| N148 |  | ✓ | ✓ | ✕ | ✓ | ✕ |
| K150 |  | ✓ | ✓ | ✓ | ✓ | ✓ |
| W152 |  | ✓ | ✓ | ✓ | ✓ | ✓ |
| R158 |  | ✓ | ✕ | ✕ | ✕ | ✓ |
| H245 |  | ✓ | ✓ | ✓ | ✕ | ✓ |
| N245 |  | ✕ | ✕ | ✕ | ✓ | ✕ |
| R246 |  | ✓ | ✓ | ✓ | ✓ | ✓ |
| S247 |  | ✓ | ✓ | ✓ | ✓ | ✓ |
| Y248 |  | ✓ | ✓ | ✓ | ✓ | ✓ |
| L249 |  | ✓ | ✓ | ✓ | ✓ | ✓ |
| T250 |  | ✕ | ✕ | ✕ | ✓ | ✕ |
| P251 |  | ✓ | ✕ | ✕ | ✕ | ✕ |
| G252 |  | ✕ | ✓ | ✕ | ✓ | ✕ |
| V252 |  | ✕ | ✕ | ✓ | ✕ | ✓ |
| D253 |  | ✓ | ✓ | ✓ | ✓ | ✓ |
| S254 |  | ✕ | ✓ | ✓ | ✓ | ✓ |
| S255 |  | ✕ | ✓ | ✓ | ✓ | ✓ |
| G257 |  | ✓ | ✓ | ✓ | ✓ | ✓ |
| W258 |  | ✕ | ✕ | ✕ | ✓ | ✕ |
| **TOT** |  | 17 | 18 | 16 | 19 | 17 |

**Table S3**

Energy contribution of the interface key residues (kcal/mol)

| **Residue** |  | int^a)^ | vdw^a)^ | eel^a)^ | pol^a)^ | tot^a)^ | **Variants** |
| --- | --- | --- | --- | --- | --- | --- | --- |
| H146 |  | 39.36 ± 0.31 | -9.09 ± 0.11 | -12.37 ± 0.18 | -5.78 ± 0.12 | 12.11 ± 0.35 | WT, BA.2, BA.2.86, JN.1, KP.2, KP.3 |
| Q146 |  | 28.75 ± 0.32 | -5.54 ± 0.13 | -62.48 ± 0.30 | -9.62 ± 0.22 | -48.89 ± 0.51 | XBB.1, XBB.1.5, HV.1 |
| N148 |  | 23.10 ± 0.31 | -2.33 ± 0.11 | -85.50 ± 0.47 | -12.75 ± 0.36 | -77.49 ± 0.27 | WT, BA.2, BA.2.86, JN.1, KP.2, KP.3 |
| W152 |  | 26.18 ± 0.35 | -12.35 ± 0.12 | 2.35 ± 0.11 | -5.64 ± 0.07 | 10.53 ± 0.37 | ALL |
| R158 |  | 29.25 ± 0.41 | -6.82 ± 0.15 | -176.37 ± 0.77 | -13.66 ± 0.91 | -167.60 ± 044 | WT, HV.1 |
| H245 |  | 38.45 ± 0.27 | -8.70 ± 0.13 | -14.21 ± 0.17 | -5.09 ± 0.16 | 10.45 ± 0.30 | WT, BA.2, XBB.1, XBB.1.5, HV.1 |
| N245 |  | 23.15 ± 0.30 | -4.44 ± 0.12 | -81.07 ± 0.37 | -7.20 ± 0.28 | -69.56 ± 0.72 | BA.2.86, JN.1, KP.2, KP.3 |
| R246 |  | 31.81 ± 0.43 | -9.91 ± 0.15 | -214.58 ± 0.75 | 10.21 ± 0.60 | -182.47 ± 0.37 | ALL |
| P251 |  | 27.54 ± 0.24 | -2.46 ± 0.10 | -4.31 ± 0.19 | -4.05 ± 0.16 | 16.72 ± 0.26 | WT |
| D253 |  | 12.95 ± 0.24 | -2.74 ± 0.17 | -42.97 ± 0.90 | -29.74 ± 0.78 | -62.50 ± 0.39 | ALL |
| W258 |  | 26.38 ± 0.34 | -7.60 ± 0.16 | -3.13 ± 0.29 | -2.49 ± 0.18 | 13.15 ± 0.51 | BA.2.86, JN.1, KP.2, KP.3 |

1. int = Internal; vdw = van der Waals; eel = electrostatic; pol = polar solvation; tot = total

**
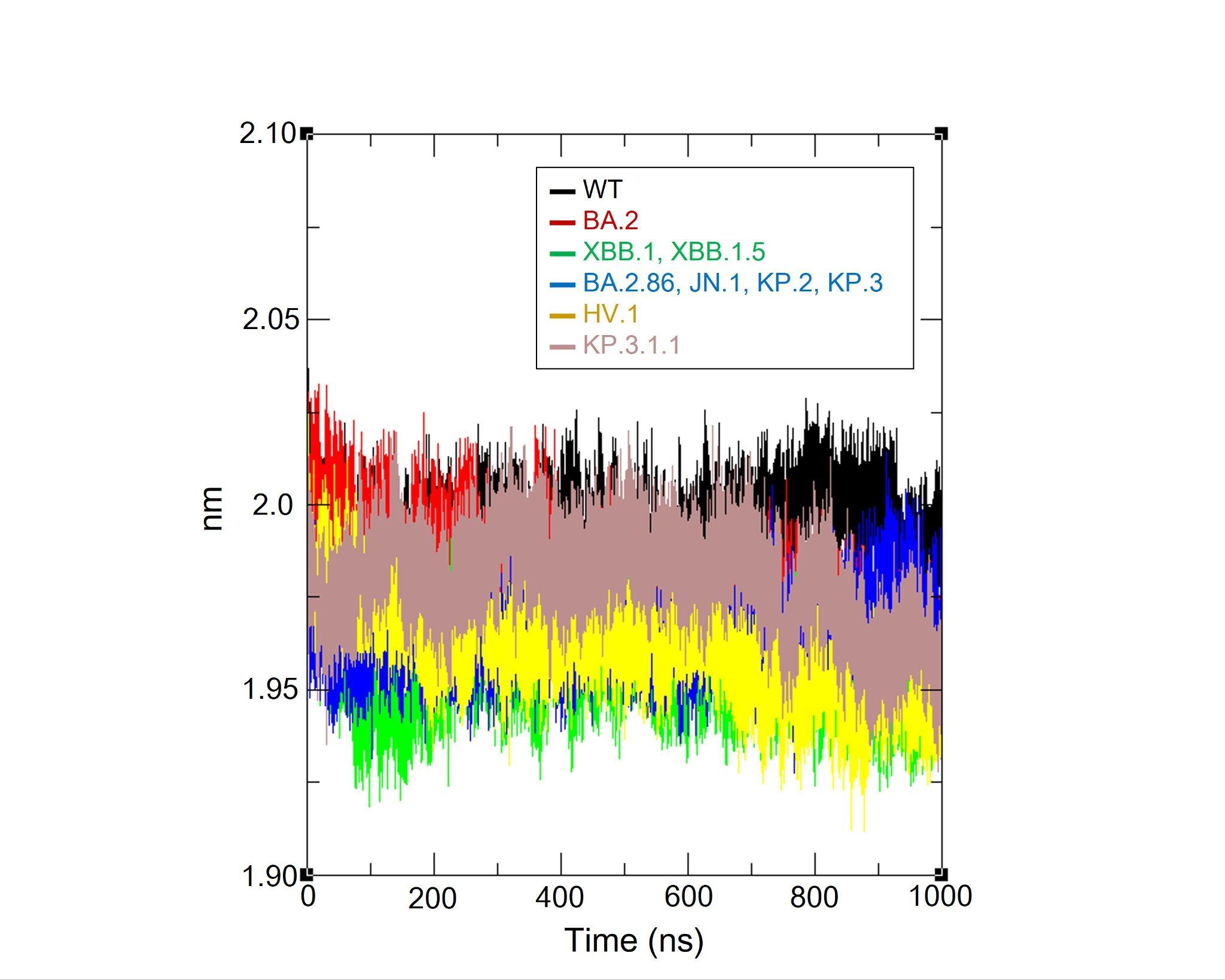
**

**A**


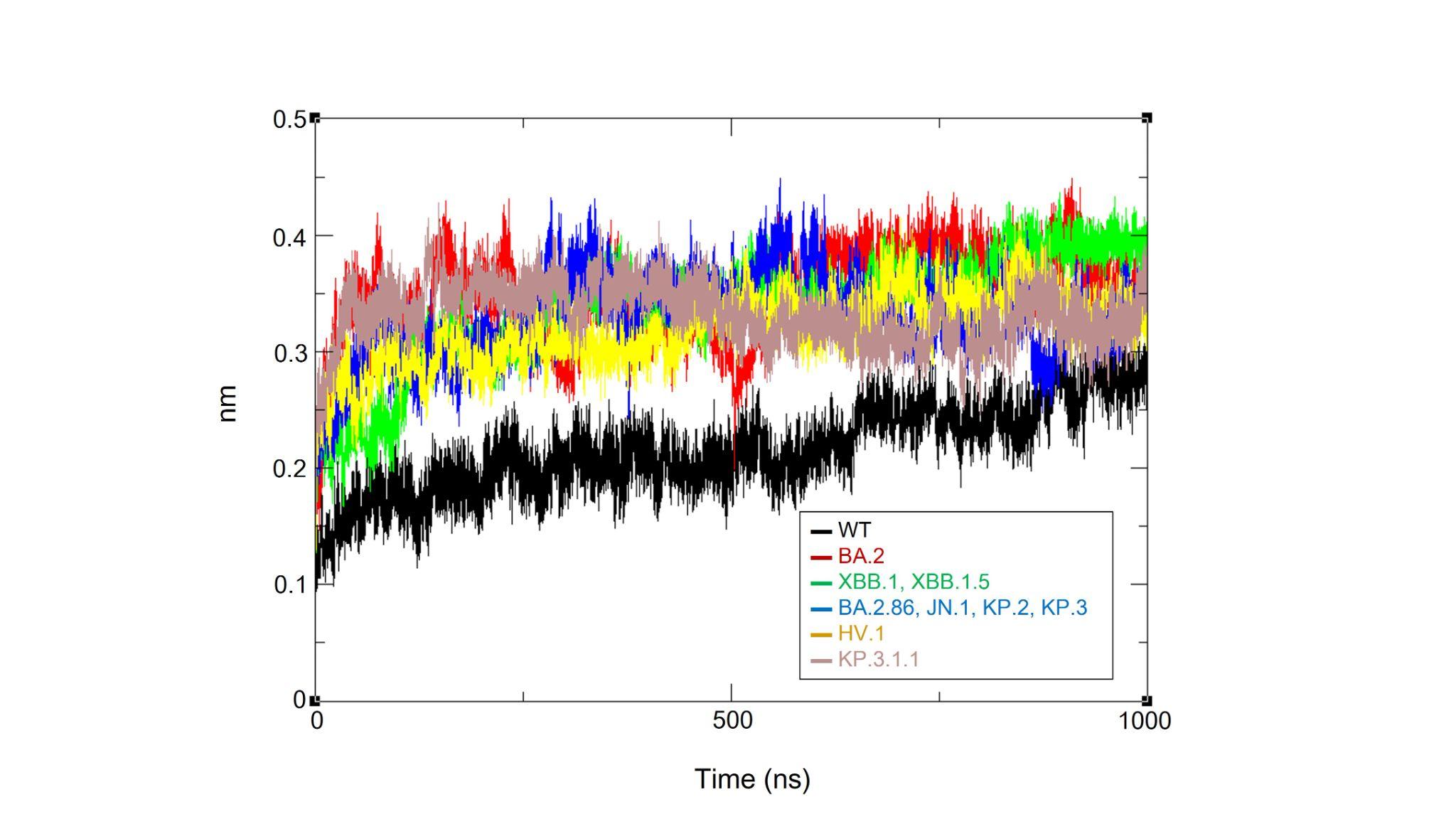


**B**

**Figure S1**

Variation of backbone radius of gyration (A) and root mean square deviation (B) during molecular dynamics simulation of the free NTD of the variants.

**
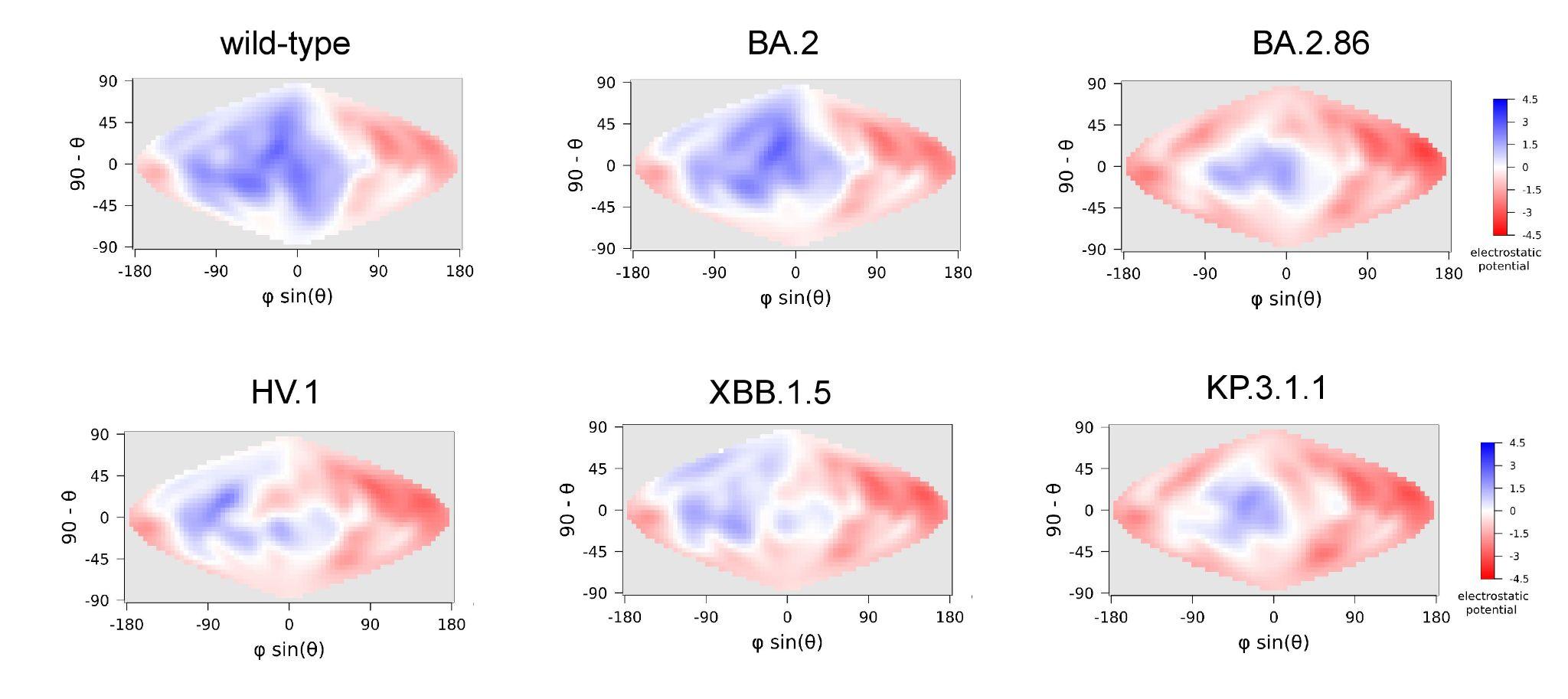
**

**Figure S2**

Two-dimensional projection of the surface electrostatic potential of the NTD variants. Domains are identically oriented. The potential color map is reported on the rights of the figure. Negative and positive charges are shown in red and blue color, respectively.


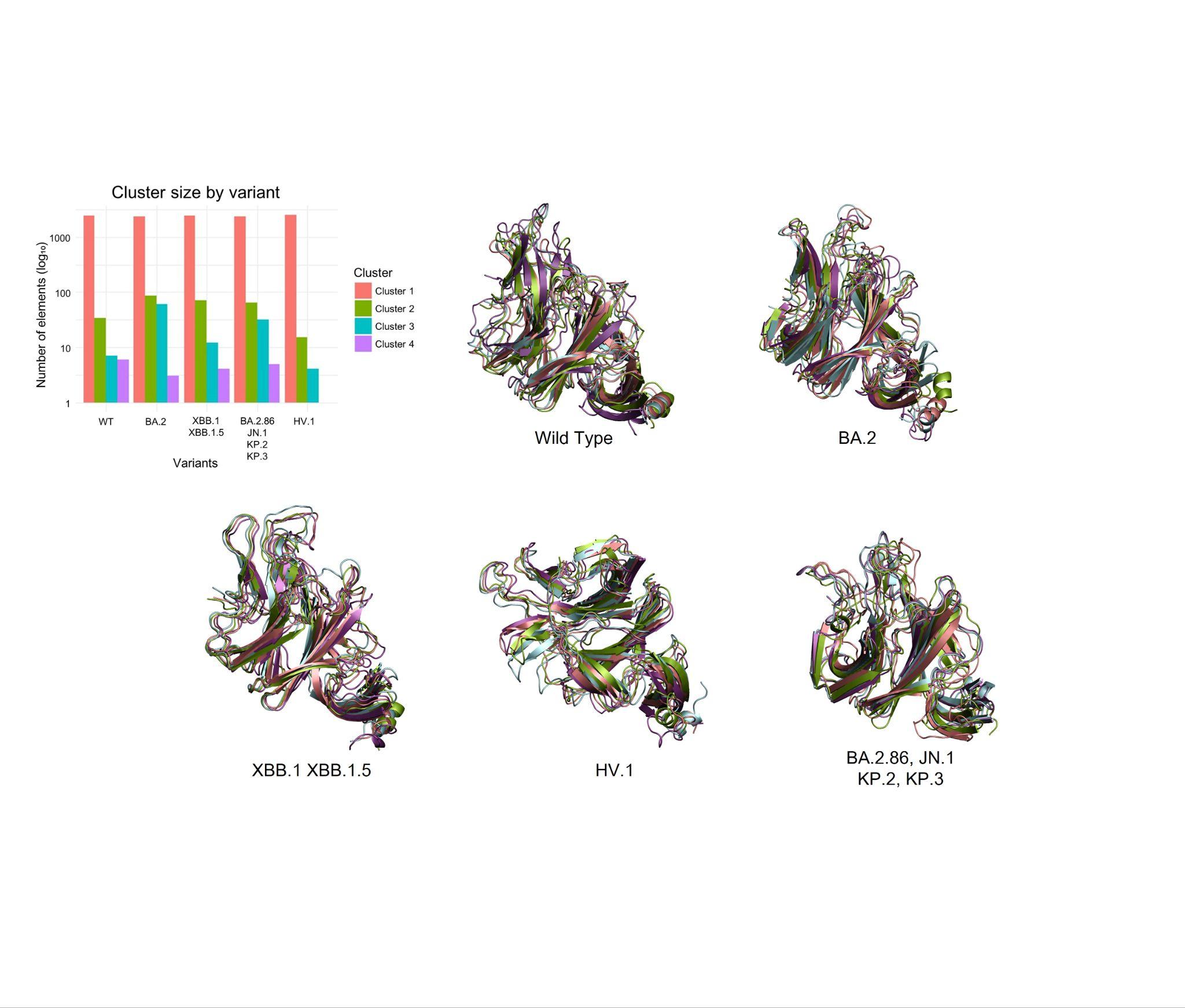


**Figure S3**

The bar chart illustrates the cluster size distribution across different variants using a logarithmic scale. The x-axis represents the variants, while the y-axis indicates the number of elements within each cluster. The legend identifies the cluster colors. The representative average structures of clusters are shown overlapped for each variant.


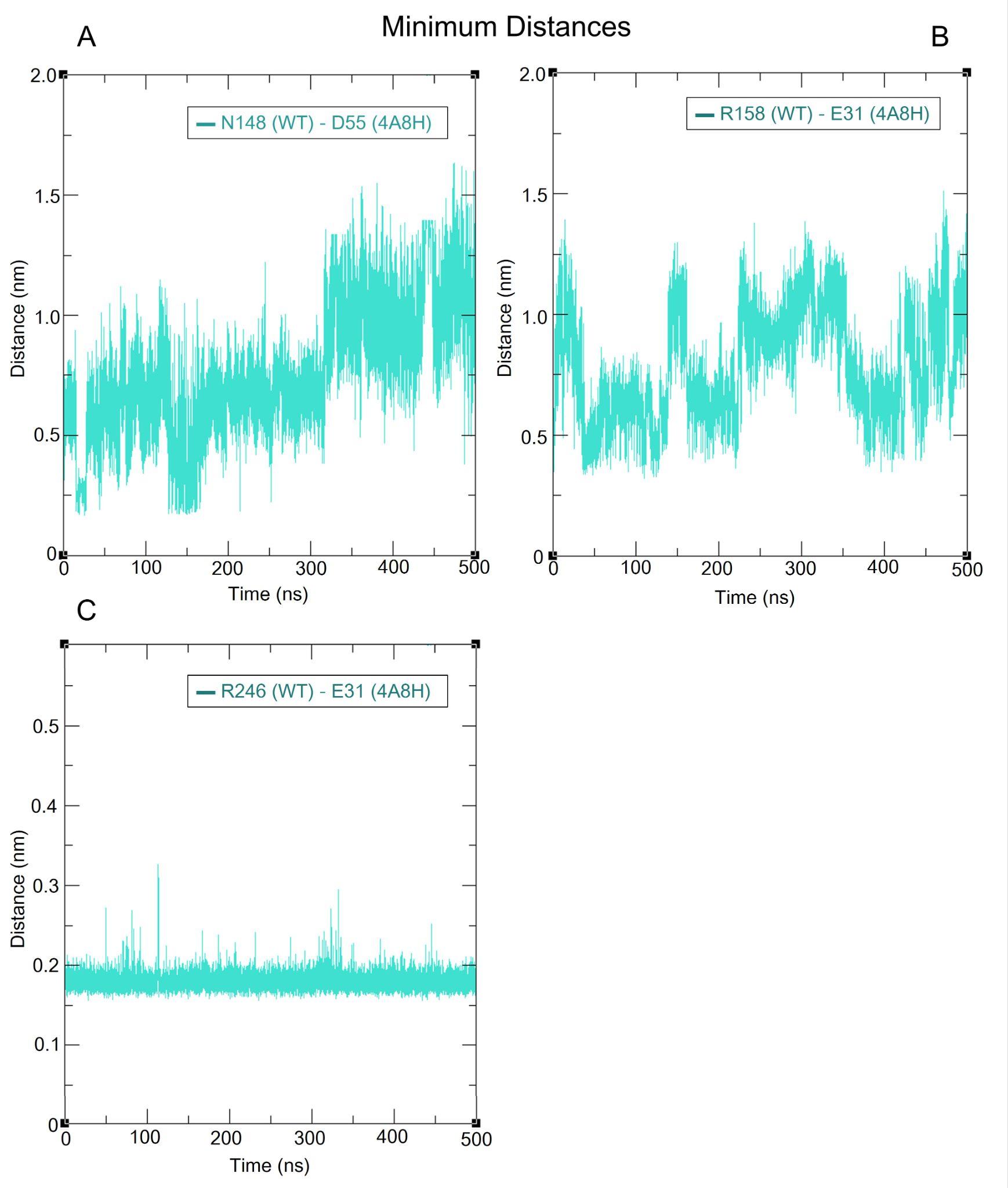


**Figure S4**

Minimum distances calculated over the entire dynamic simulation time between pairs of residues in the WT-4A8 complex. **A)** Minimum distance between N148 of the NTD of WT and D55 of 4A8H. **B)** Minimum distance between R158 of the WT-NTD and E31 of 4A8H. **C)** Minimum distance between R246 of the WT-NTD and E31 of 4A8H.


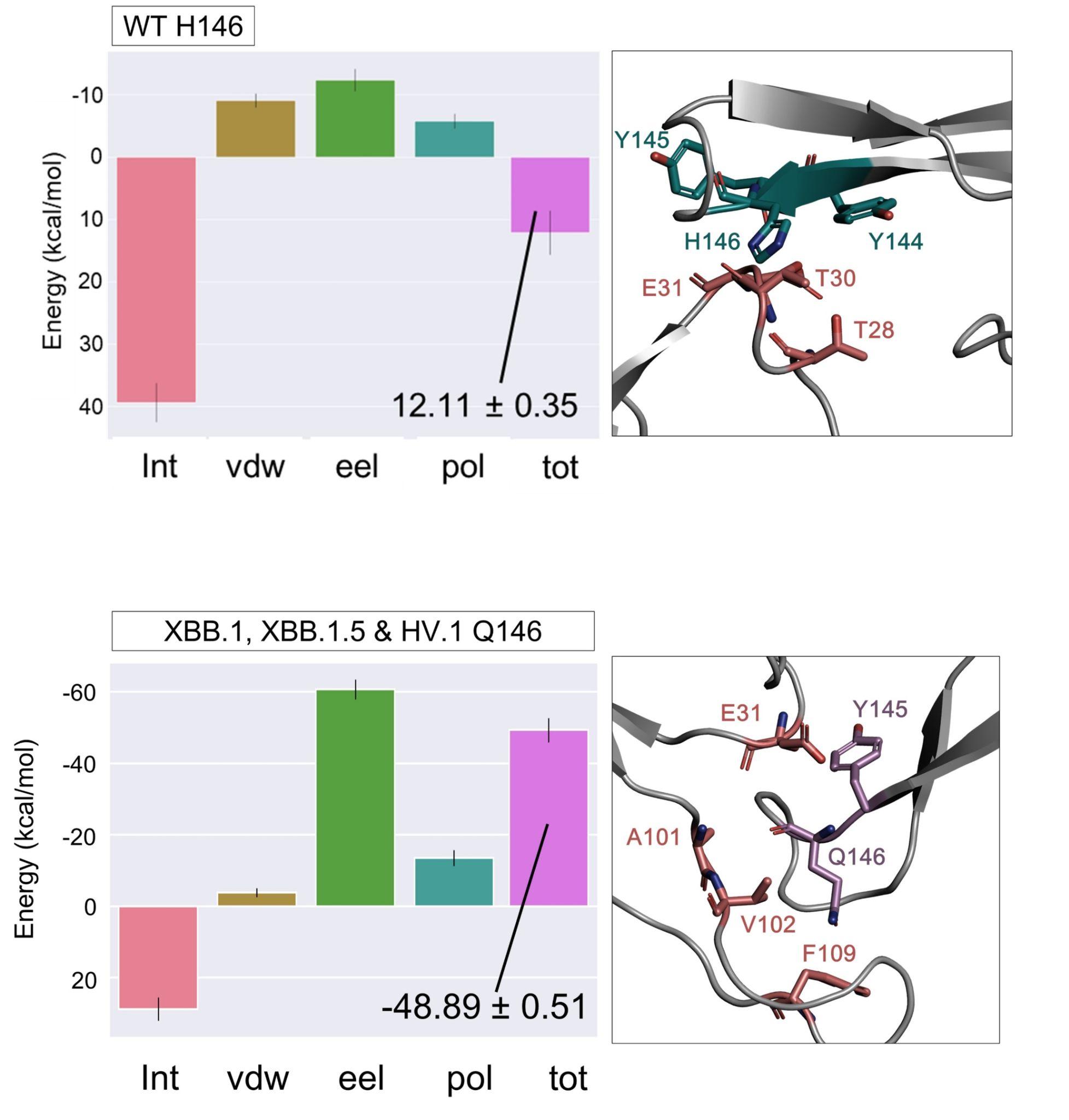


**Figure S5**

Bar plots of the decomposition of energy components (kcal/mol) of the residue in the position 146 of WT, XBB.1, XBB.1.5 and HV.1. Int = Internal energy contributions; vdw = van der Waals energy contributions; eel = electrostatic energy contributions; pol = polar solvation free energy contributions; tot = total free energy contributions (sum of all). At the side of the bar plots, the structural representation of residue interactions is reported according to the clusters 1-3-4 and 1-2-3-4, respectively, for the WT and the variants. Cyan, red-salmon and magenta labels indicate WT-NTD, 4A8H and HV.1-NTD residues, respectively.

**
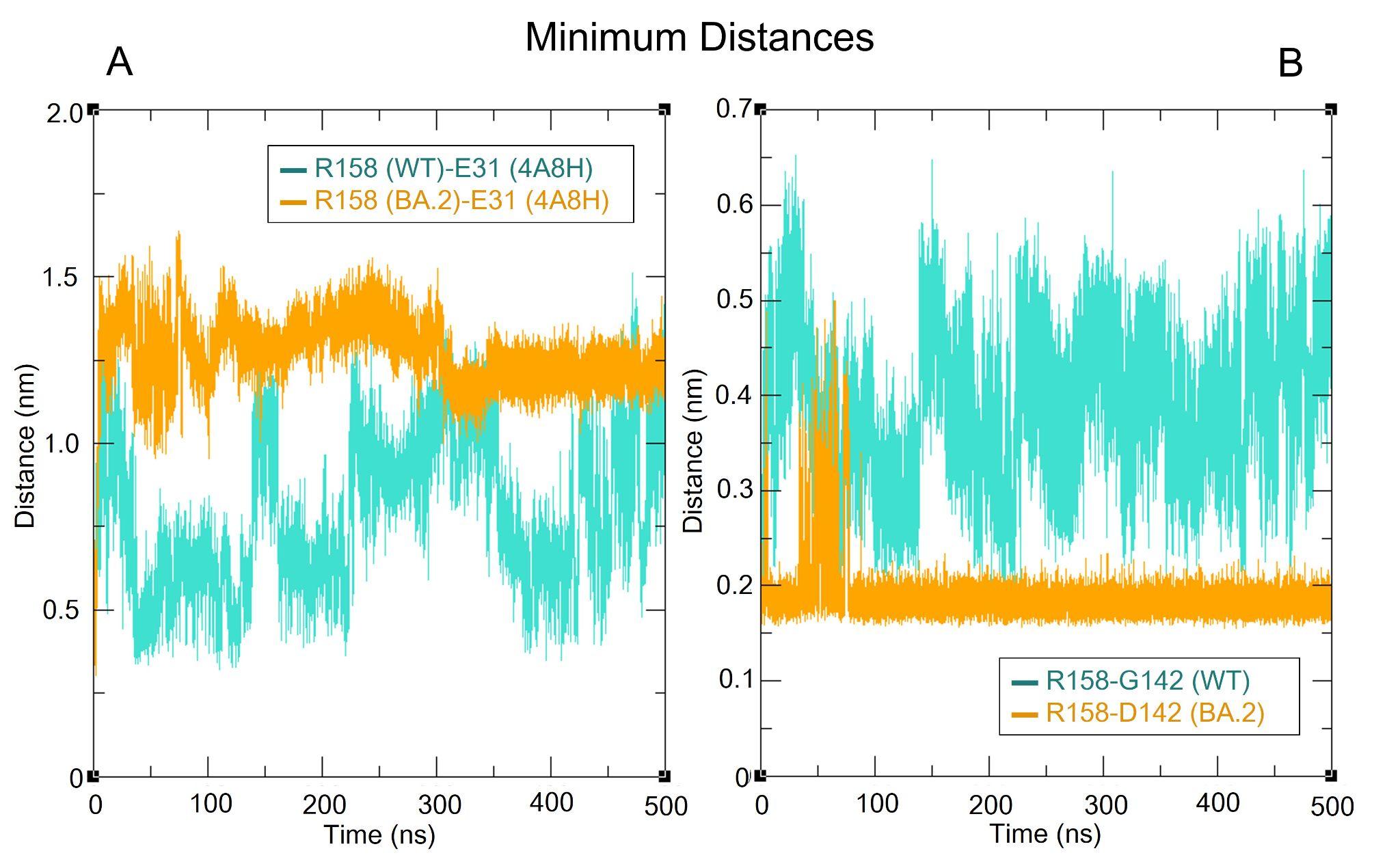
**

**Figure S6**

**A)** Comparison of the minimum distance between R158 of the WT and BA.2 with 4A8H E31. **B)** Comparison of the minimum distance between R158 and G142 in the WT, with R158 and D142 in BA.2.


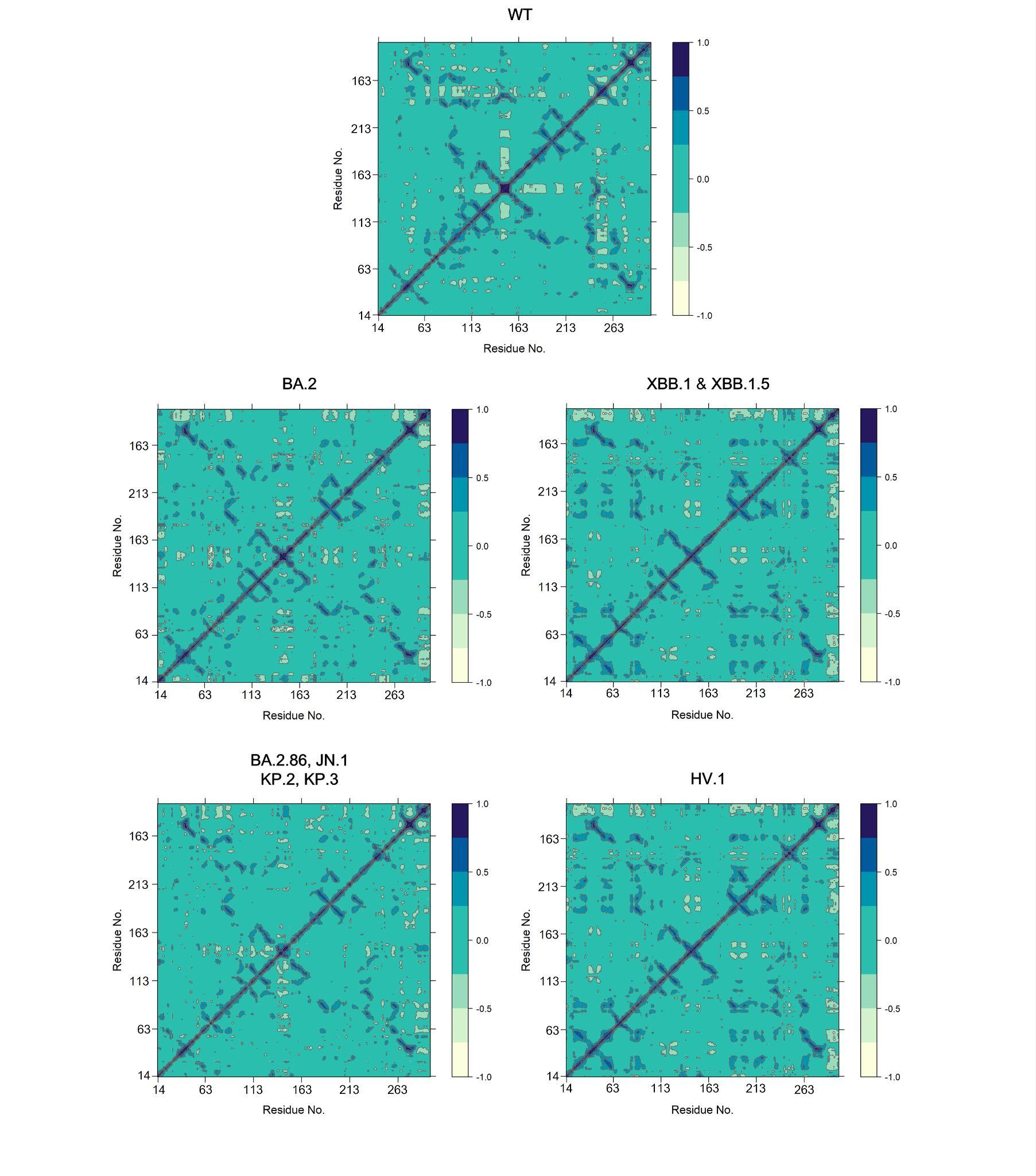


**Figure S7**

Dynamic cross-correlation matrices (DCCM) for Cα atom pairs of the NTDs from all variants calculated from the complexes last 250 ns simulation. The correlation color scale is displayed to the right of each graph, labeled with the corresponding variant.


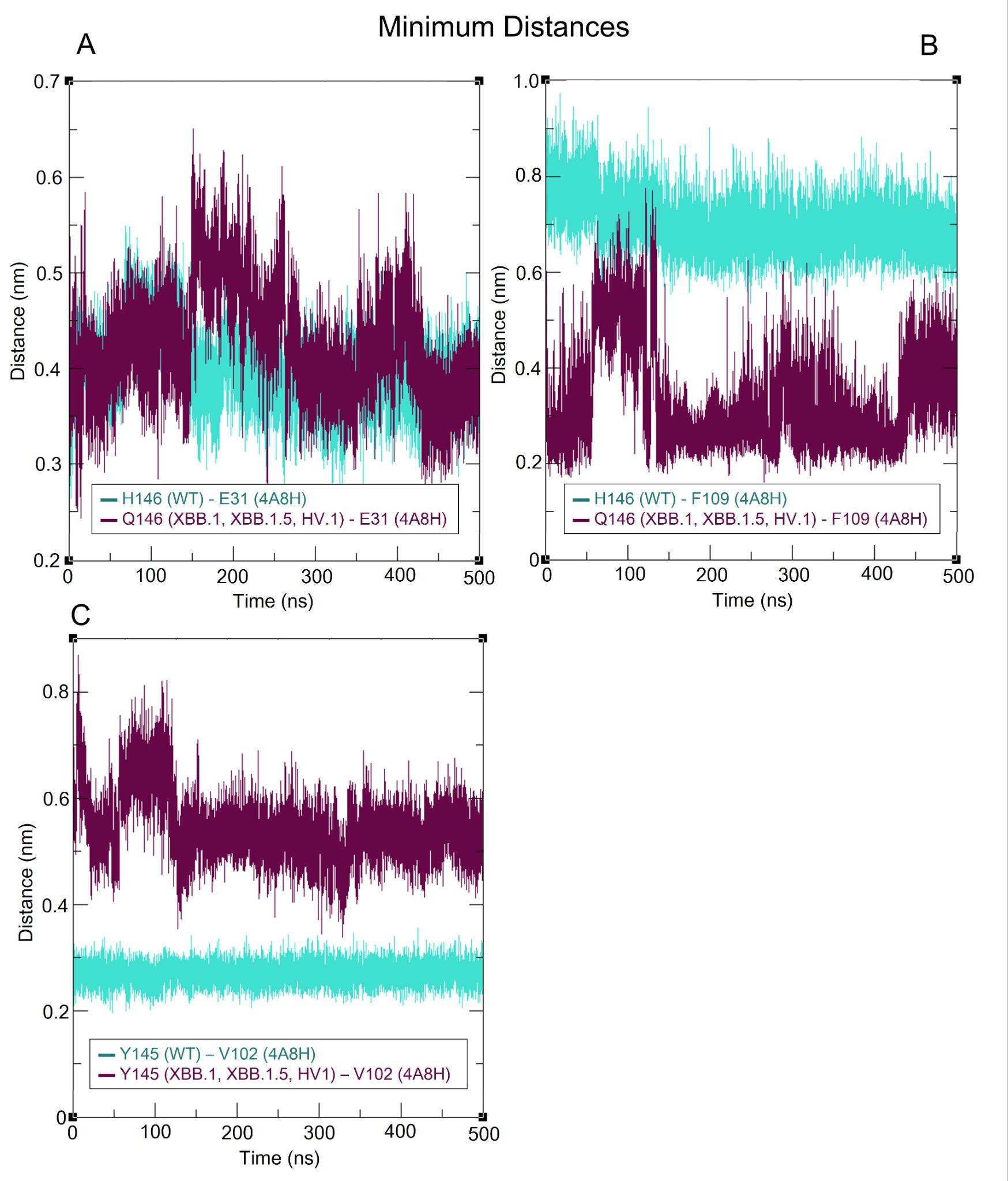


**Figure S8**

**A)** Comparison of the minimum distance between H146 (WT) and Q146 (XBB.1, XBB.1.5, HV.1) with E31of 4A8H. **B)** Comparison of the minimum distance between H146 (WT) and Q146 (XBB.1, XBB.1.5, HV.1) with F109 of 4A8H. **C)** Comparison of the minimum distance between NTD Y145 and V102 of 4A8H in the WT, XBB.1, XBB.1.5, and HV.1 caused by the deletion of Y144.

**
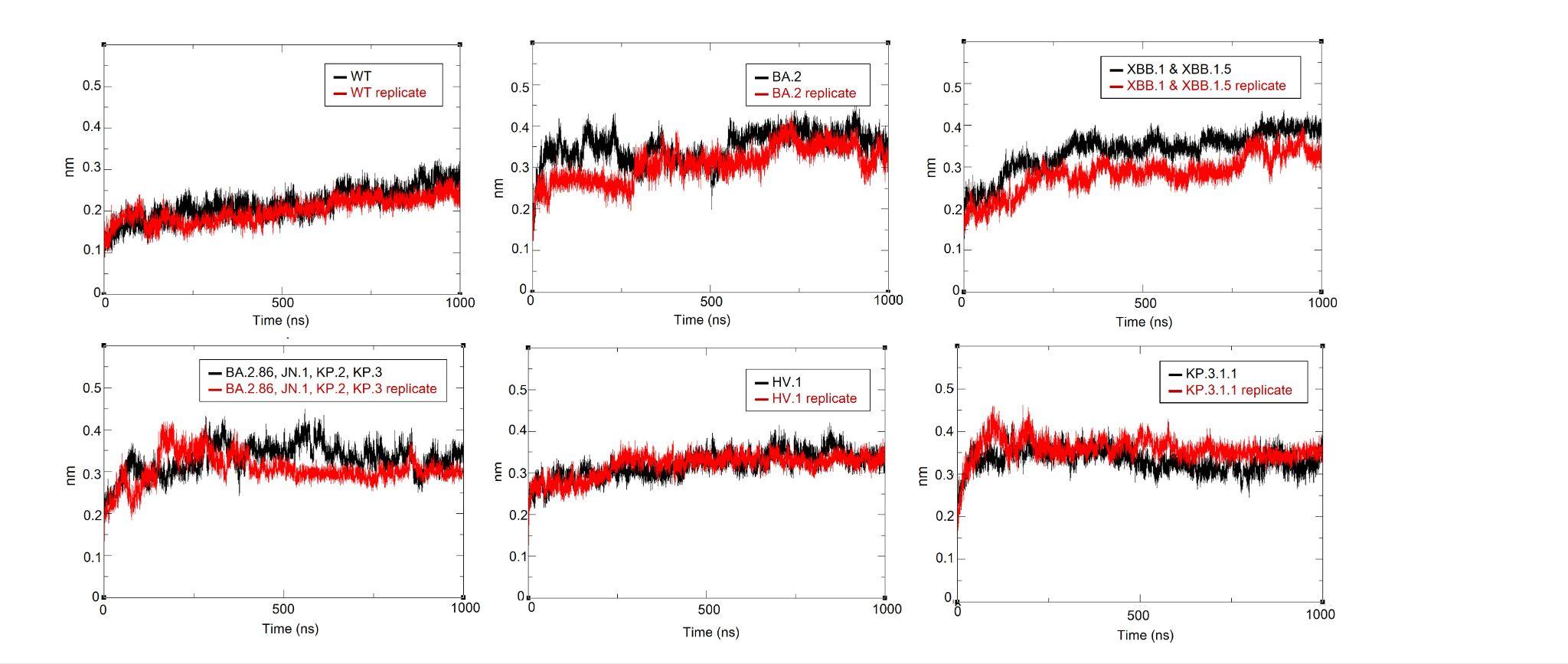
**

**a**

**
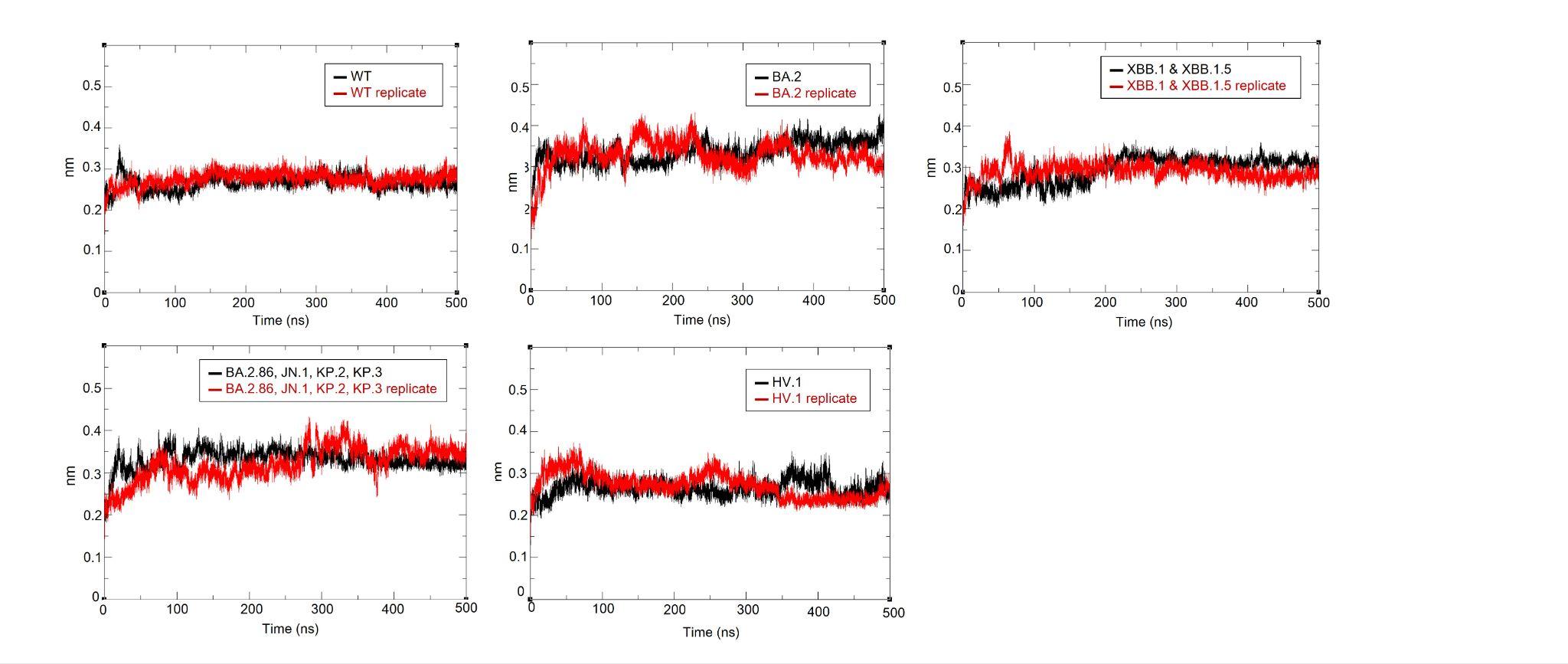
**

**b**

**Figure S9**

**A)** Comparison of the root mean square deviation of free NTDs and **B)** NTDs calculated from complex simulations with their respective replicates.
